# Malonyl-acyl carrier protein decarboxylase activity promotes fatty acid and cell envelope biosynthesis in Proteobacteria

**DOI:** 10.1016/j.jbc.2021.101434

**Published:** 2021-11-18

**Authors:** Sarah G. Whaley, Christopher D. Radka, Chitra Subramanian, Matthew W. Frank, Charles O. Rock

**Affiliations:** Department of Infectious Diseases, St Jude Children’s Research Hospital, Memphis, Tennessee, USA

**Keywords:** *Escherichia coli*, fatty acid synthesis, malonyl-ACP decarboxylase, FabH, GlmU, ACP, acyl carrier protein, ECA, enterobacterial common antigen, FabH, 3-ketoacyl-ACP synthase III, FASII, type II bacterial fatty acid synthesis system, GlmU, glucosamine-1-phosphate *N*-acetyl transferase/*N*-acetyl-glucosamine-1-phosphate uridylyltransferase, GNAT, GCN5-related *N*-acetyl transferase, Mad, malonyl-ACP decarboxylase, LPS, lipopolysaccharide, PG, peptidoglycan, SAXS, small angle X-ray scattering

## Abstract

Bacterial fatty acid synthesis in *Escherichia coli* is initiated by the condensation of an acetyl-CoA with a malonyl-acyl carrier protein (ACP) by the β-ketoacyl-ACP synthase III enzyme, FabH. *E. coli* Δ*fabH* knockout strains are viable because of the *yiiD* gene that allows FabH-independent fatty acid synthesis initiation. However, the molecular function of the *yiiD* gene product is not known. Here, we show the *yiiD* gene product is a malonyl-ACP decarboxylase (MadA). MadA has two independently folded domains: an amino-terminal *N*-acetyl transferase (GNAT) domain (MadA^N^) and a carboxy-terminal hot dog dimerization domain (MadA^C^) that encodes the malonyl-ACP decarboxylase function. Members of the proteobacterial Mad protein family are either two domain MadA (GNAT-hot dog) or standalone MadB (hot dog) decarboxylases. Using structure-guided, site-directed mutagenesis of MadB from *Shewanella oneidensis*, we identified Asn45 on a conserved catalytic loop as critical for decarboxylase activity. We also found that MadA, MadA^C^, or MadB expression all restored normal cell size and growth rates to an *E. coli* Δ*fabH* strain, whereas the expression of MadA^N^ did not. Finally, we verified that GlmU, a bifunctional glucosamine-1-phosphate *N*-acetyl transferase/*N*-acetyl-glucosamine-1-phosphate uridylyltransferase that synthesizes the key intermediate UDP-GlcNAc, is an ACP binding protein. Acetyl-ACP is the preferred glucosamine-1-phosphate *N*-acetyl transferase/*N*-acetyl-glucosamine-1-phosphate uridylyltransferase substrate, in addition to being the substrate for the elongation-condensing enzymes FabB and FabF. Thus, we conclude that the Mad family of malonyl-ACP decarboxylases supplies acetyl-ACP to support the initiation of fatty acid, lipopolysaccharide, peptidoglycan, and enterobacterial common antigen biosynthesis in Proteobacteria.

Bacterial type II fatty acid synthesis (FASII) is catalyzed by a collection of conserved enzymes that supply fatty acids for membrane phospholipid synthesis and has been the subject of intense study for decades (1). Initially, FASII was thought to be initiated by an acetyl-CoA:acyl carrier protein (ACP) transacylase to generate an acetyl-ACP primer and an enzyme that catalyzes this reaction was purified (2). In this original hypothesis, the acetyl-ACP is then used by the elongation-condensing enzymes (FabB or FabF) to initiate cycles of elongation (2, 3, 4). In 1987, an enzyme was discovered that catalyzes the condensation of acetyl-CoA with malonyl-ACP in *Escherichia coli* to initiate FASII (5), and the *fabH* gene encoding this activity was identified in 1992 (6). FabH homologs are widely distributed and are the major enzymes responsible for the initiation of FASII (1) and are targets for antibiotic drug discovery (7, 8). FabH is a ping-pong enzyme and also catalyzes acetyl-CoA:ACP transacylation albeit at a much lower rate than the condensation reaction (9), suggesting that FabH may be responsible for the transacylation activity noted previously (2). In *E. coli*, *fabH* was thought to be an indispensable gene because knockout strains could not be derived (10). These experiments were performed in a strain with two mutations (*relA1 and spoT1*) that perturb the regulation of the alarmone ppGpp. However, Δ*fabH* and Δ*spoT* are synthetically lethal, and Δ*fabH* strains are recovered in either a WT or *relA1* background (11). Although these Δ*fabH* strains grew significantly slower and had a smaller cell size than the WT, this result meant that there was another enzyme that could partially substitute for FabH. Sanyal *et al.* (12) identified this gene as *yiiD*, but the catalytic activity of YiiD remains unknown.

This study identifies YiiD as a malonyl-ACP decarboxylase (MadA) that produces acetyl-ACP. MadA has an amino terminal *N*-acetyl transferase (GNAT) domain (MadA^N^) fused to a carboxy terminal hot dog-fold domain (MadA^C^) encoding the malonyl-ACP decarboxylase function. MadB is a standalone hot dog malonyl-ACP decarboxylase from *Shewanella oneidensis*, and structure-guided mutagenesis identifies Asn45 as a key catalytic residue. MadA, MadA^C^, or MadB expression restores cell size and growth rate to an *E. coli* Δ*fabH* strain, whereas the expression of MadA^N^ does not. We verify that GlmU (glucosamine-1-phosphate *N*-acetyl transferase/*N*-acetyl-glucosamine-1-phosphate uridylyltransferase) is an ACP binding protein and show that acetyl-ACP is the preferred GlmU substrate for the synthesis of UDP-GlcNAc. Thus, the Mad family of malonyl-ACP decarboxylases supplies acetyl-ACP to support the initiation of FASII, lipopolysaccharide (LPS), peptidoglycan (PG) and enterobacterial common antigen (ECA) biosynthesis in Proteobacteria.

## Results

### MadA (YiiD) is a malonyl-ACP decarboxylase

**Figure 1:**
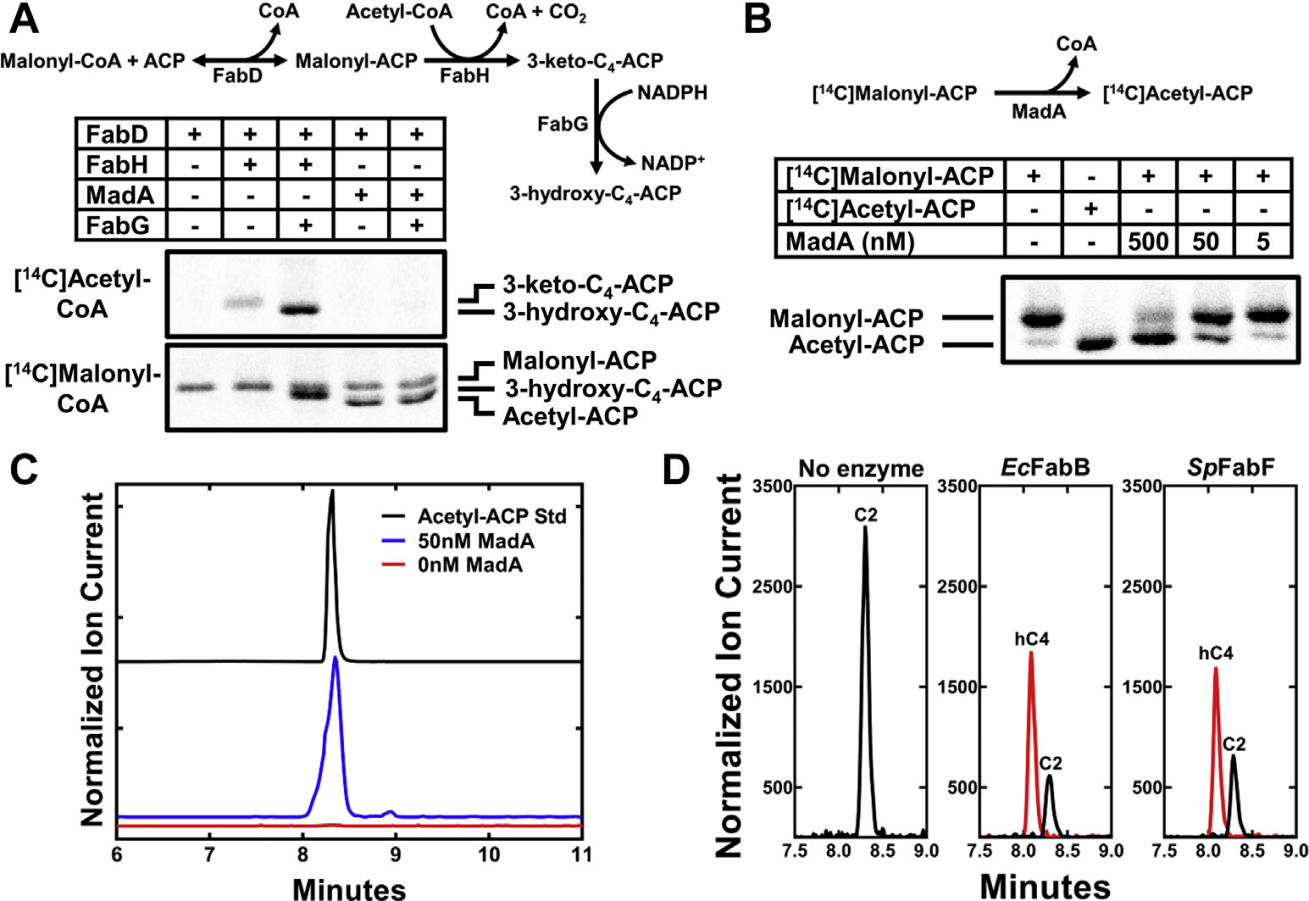
**The *yiiD* gene encodes a malonyl-ACP decarboxylase (Mad).***A*, FabH-condensing enzyme assay using either [1-^14^C]acetyl-CoA or [2-^14^C]malonyl-CoA as the labeled substrate. The coupled reaction scheme is shown above. The assays were analyzed using urea gel electrophoresis to separate ACP thioesters (13). The attached acyl chains are labeled. *B*, malonyl-ACP decarboxylase activity of MadA. [2-^14^C]Malonyl-ACP and [1-^14^C]acetyl-ACP were prepared with AcpS. The conversion of malonyl-ACP to acetyl-ACP was catalyzed by MadA alone. *C*, mass spectrometry identifies acetyl-ACP as the MadA product. The assays were performed with nonradiolabeled substrates plus MadA. The samples hydrolyzed with Asp-N protease and acetyl-ACP was identified by LC-MS/MS using the *m/z* = 716.3/303.2 (Q1/Q3) masses characteristic for acetyl-ACP. *D*, the elongation-condensing enzymes use acetyl-ACP. The assays were performed with acetyl-ACP, malonyl-CoA, FabD, FabG, and NADPH and either *E. coli* FabB or *S. pneumoniae* FabF elongation-condensing enzymes. The 3-hydroxybutyryl-ACP arising from FabB or FabF condensation of acetyl-ACP (C2, *black*) with malonyl-ACP was detected by LC-MS/MS using the *m/z* = 760.3/347.2 (Q1/Q3) masses characteristic for 3-hydroxybutyryl-ACP (hC4, *red*). ACP, acyl carrier protein; FabH, 3-ketoacyl-ACP synthase III.

We first tested whether the purified MadA (Fig. S1*A*) catalyzed a condensing enzyme reaction that replaced FabH function using a coupled enzyme assay according to the scheme outlined in Figure 1*A*. FabD was used to produce malonyl-ACP that was converted to 3-ketobutyryl-ACP by FabH in the presence of acetyl-CoA. The 3-ketobutyryl-ACP product is not an abundant product, so FabG was used in a coupled reaction to reduce 3-ketobutyryl-ACP to the stable 3-hydroxybutyryl-ACP (13, 14, 15). The samples were separated on urea gels that resolve the different ACP thioesters based on their ability to stabilize ACP conformation (13, 14, 15). FabH formed 3-ketobutyryl-ACP and 3-hydroxybutyryl-ACP in the coupled enzyme system from [^14^C]acetyl-CoA, but MadA did not (Fig. 1*A*), showing that MadA does not carry out a FabH-like condensing enzyme reaction. In addition, MadA is not an acetyl-CoA:ACP acetyltransferase because [^14^C]acetyl-ACP was not detected in these experiments. When [2-^14^C]malonyl-CoA was used as substrate, [^14^C]malonyl-ACP was formed and converted by FabH to 3-hydroxybutyryl-ACP (Fig. 1*A*). However, [2-^14^C]malonyl-ACP was converted to a different product by MadA that appeared to be [^14^C]acetyl-ACP based on its electrophoretic mobility (Fig. 1*A*).

Assays containing only MadA and [2-^14^C]malonyl-ACP verified that MadA was necessary and sufficient for acetyl-ACP formation (Fig. 1*B*). The [2-^14^C]malonyl-ACP and [^14^C]acetyl-ACP standards were synthesized using AcpS and apo-ACP to create the labeled acyl-ACPs from their respective labeled acyl-CoAs. Commercial [2-^14^C]malonyl-CoA is contaminated with a few percent [^14^C]acetyl-CoA, and AcpS prefers acetyl-CoA over malonyl-CoA (16) resulting in the unavoidable contamination of the [2-^14^C]malonyl-ACP substrate preparation with a detectable level of [^14^C]acetyl-ACP product (Fig. 1*B*). MadA addition results in the dose-dependent decarboxylation of [2-^14^C]malonyl-ACP to [^14^C]acetyl-ACP (Fig. 1*B*). The MadA product was verified as acetyl-ACP by mass spectrometry (Fig. 1*C*). The reactions were performed with nonradioactive substrates, the samples were acid precipitated, and the pellets hydrolyzed with Asp-N protease. This process liberates a tripeptide harboring the prosthetic group and attached acyl chains that were then separated and detected by LC-MS/MS (17, 18, 19). Acetyl-ACP was not detected in assays without MadA and was robustly detected in assays containing MadA (Fig. 1*C*). Acetyl-ACP is readily used by *E. coli* FabB or *Streptococcus pneumoniae* FabF-condensing enzymes (Fig. 1*D*), confirming that acetyl-ACP is used to initiate FASII by these enzymes. These data verify acetyl-ACP as the MadA product and identify the product of the *yiiD* gene as a Mad. Sanyal *et al.* called *yiiD fabY* (12); however, the FabY name was designated as a *bona fide* FabH-condensing enzyme homolog from *Pseudomonas aeruginosa* in 2012 (20). Now that the function of YiiD is known, we designate the *yiiD* gene as *madA*.

**Figure 2:**
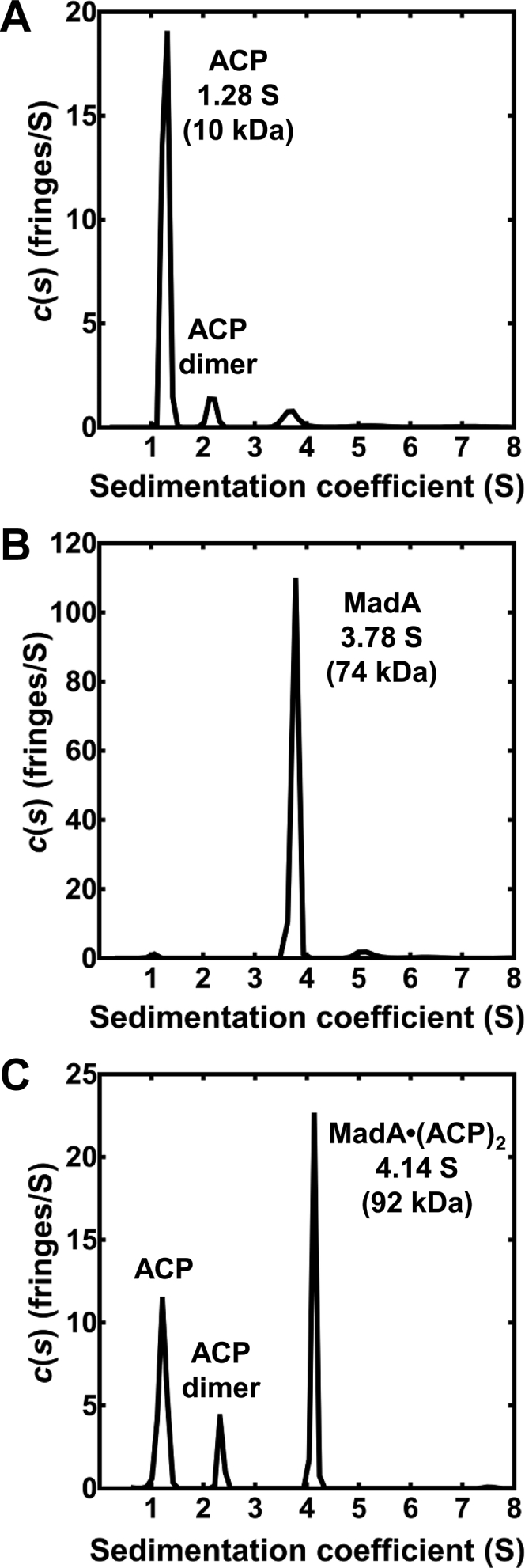
**Sedimentation analysis of ACP binding to MadA.***A*, sedimentation analysis of ACP. Reducing agents were not used in these studies and ACP-disulfide dimers are detected. *B*, sedimentation analysis of MadA shows that it exists as a dimer. *C*, sedimentation analysis of a MadA-ACP mixture. Complex formation is detected, and the *s*_*20*_ value of the peak is consistent with two ACPs bound to each MadA dimer (MadA·(ACP)_2_). Sedimentation equilibrium parameters are listed in Table S1. ACP, acyl carrier protein; Mad, malonyl-ACP decarboxylase.

Analytical ultracentrifugation verified that MadA is a dimeric ACP-binding protein (Table S1). ACP is a monomer (Fig. 2*A*), and MadA was confirmed as a dimer by sedimentation equilibrium analysis (Fig. 2*B*). The analysis of MadA plus ACP resulted in the appearance of a new 92-kDa protein species that corresponds in molecular weight to a MadA·(ACP)_2_ complex (Fig. 2*C*) meaning that there are two ACP-binding sites per MadA dimer.

### MadA has two independent domains

**Figure 3:**
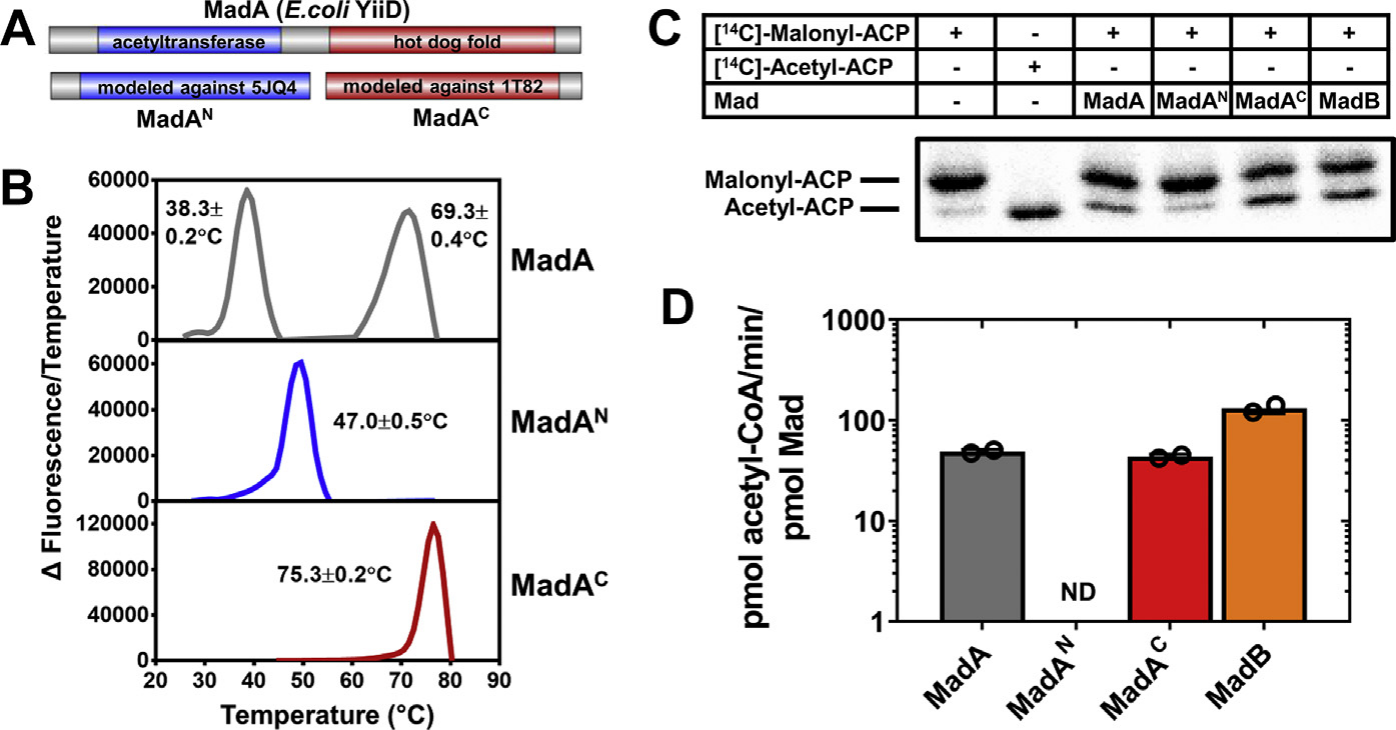
**Domain structure of MadA and MadB.***A*, bioinformatic analysis predicts MadA has a two domain architecture consisting of a MadA^N^ amino terminal domain that is highly related to GNAT acetyltransferases (PDB ID: 5JQ4) fused to a carboxyl terminal MadA^C^ hot dog domain related to proteins in Pfam_09500 (PDB ID: 1T82). *B*, thermal denaturation temperatures for MadA, MadA^N^, and MadA^C^. Representative first derivatives of the denaturation curves that were used to identify the mean temperature ±SEM from triplicate experiments. *C*, malonyl-ACP decarboxylase activity of MadA, MadA^N^, MadA^C^, and MadB assessed by the gel electrophoresis assay. *D*, specific activities of MadA, MadA^C^, and MadB using the malonyl-CoA decarboxylase assay were 49.2 ± 1.7, 43.8 ± 1.6, and 132 ± 10.73 pmol/min/pmol proteins, respectively. MadA^N^ malonyl-CoA decarboxylase activity was not detected (<6.9 × 10^−5^ pmol/min/pmol protein). ACP, acyl carrier protein; GNAT, GCN5-related N-acetyl transferase; Mad, malonyl-ACP decarboxylase.

The bioinformatic analysis of MadA indicates that it is composed of two distinct protein domains, MadA^N^ and MadA^C^ (Fig. 3*A*). The amino terminal MadA^N^ domain has distinct homology to protein acetyltransferases in the GNAT superfamily. These proteins typically use acetyl-CoA to acylate primary amines such as lysine amino groups on proteins (21). Specifically, MadA^N^ belongs to the Acetyltransf_1 protein family (Pfam_00583) and is most closely related to the PDB entry for a GNAT acetyltransferase of unknown function from *Staphylococcus aureus* (PDB ID: 5JQ4). Mapping the MadA^N^ sequence onto this acetyltransferase structure suggests that the acetyltransferase domain of MadA^N^ lies between residues 19 and 162 (Fig. 3*A*). The carboxy terminal domain has a predicted hot dog fold associated with enzymes that carry out thioesterase, hydratase, or dehydratase reactions (22, 23, 24). The MadA^C^ domain belongs to Pfam_09500, which consists of highly related hot dog proteins that are widely expressed in Proteobacteria and are annotated as thioesterases. Mapping the MadA^C^ sequence onto the structure of a Pfam_09500 member from *S. oneidensis* (PDB ID: 1T82) suggests that the MadA^C^ domain encompasses residues 171 to 314 (Fig. 3*A*). These two domains are connected by a 10 amino acid region of low complexity containing three proline and five threonine residues, indicating that MadA consists of two independently folded domains connected by a flexible spacer. We constructed MadA^N^ and MadA^C^ recombinant proteins as outlined in Figure 3*A*. MadA^N^ migrated as a monomer (Fig. S1*B*) and the MadA^C^ migrated as a dimer (Fig. S1*C*) by gel filtration chromatography. Sedimentation equilibrium analysis confirmed that MadA^N^ is a monomer (Fig. S2*A*) and MadA^C^ is a dimer (Fig. S2*B*) (Table S1). Thus, MadA^C^ encodes the MadA dimerization domain.

The thermal denaturation profiles of the recombinant proteins showed MadA has two distinct structural transitions at 38 °C and 69 °C (Fig. 3*B*). This unique denaturation pattern supports the idea that MadA consists of two independently folded and noninteracting protein domains. Purified MadA^N^ was a compactly folded protein with a denaturation temperature of 47 °C and MadA^C^ denaturated at 75 °C (Fig. 3*B*). The isolated individual domains are more stable than when they are tethered together in MadA. The individual domains were tested for malonyl-ACP decarboxylase activity. MadA^N^ did not catalyze the malonyl-ACP decarboxylase reaction, whereas MadA^C^, like MadA, is an active malonyl-ACP decarboxylase (Fig. 3*C*). Thus, MadA^C^ is an independently folded hot dog dimerization domain that is necessary and sufficient for malonyl-ACP decarboxylase activity.

### MadB malonyl-ACP decarboxylases

There are 740 sequences containing the MadA^C^ domain architecture in Pfam_09500. Among those, 675 occur in Proteobacteria, 20 are in Verrucomicrobia, 11 are found in Firmicutes, and the remaining 34 are scattered among 12 additional phyla (Table S2). The most common protein architecture is a standalone MadA^C^ domain occurring in 470 of the 740 sequences. We call the standalone members of Pfam_09500 MadB. There are 270 of the 740 sequences with the MadA^C^ domain fused to an amino terminal domain belonging to a member of the GNAT family of acetyltransferases. The *E. coli* MadA is a fusion of the hot dog domain to an Acetyltransf_1 GNAT domain (Pfam_00583) and is the most common two-domain organization occurring in 214 of the 270 sequences. The MadA proteins occur almost exclusively in the Gammaproteobacteria.

The prediction that MadB proteins in Pfam_09500 are malonyl-ACP decarboxylases was validated by characterizing the MadB of *S. oneidensis*, the only Mad protein with a high-resolution X-ray structure (PDB ID: 1T82) (25). The 18-kDa recombinant MadB purified as a dimer by gel filtration chromatography (Fig. S1*D*), and the dimeric state of MadB was confirmed by sedimentation equilibrium analysis (Fig. S2*C*). MadB was a stable protein with a denaturation temperature of 71 ± 0.2 °C. MadB possessed comparable malonyl-ACP decarboxylase activity with that of MadA^C^ (Fig. 3*C*). Thus, MadB of *S. oneidensis*, and by inference all MadA and MadB members of Pfam_09500, are malonyl-ACP decarboxylases.

The gel assay for Mad activity is time consuming and a required reagent, [2-^14^C]malonyl-ACP, is difficult to synthesize and spontaneously degrades to [^14^C]acetyl-ACP over time (Fig. S3*A*). Usually, acyl-ACP is stored at pH 6 to 7 to minimize hydrolysis of the base-labile thioester bond. However, the conversion of malonyl-ACP to acetyl-ACP is an additional stability problem that is accelerated by low pH, heat, and time (Fig. S3*A*). This stability is a characteristic of malonyl-ACP because malonyl-CoA does not degrade to acetyl-CoA under the same conditions (Fig. S3*B*). The activities of ACP-dependent enzymes are often assayed using acyl-CoAs as surrogate, low-affinity substrates (26). Therefore, we developed a Mad assay using the substrate analog malonyl-CoA as a reliably quantitative approach to measuring Mad activity (Fig. 3*D*). Malonyl-CoA is a substrate for the Mad proteins with a Km >5 mM and a correspondingly lower enzyme specific activity than with malonyl-ACP. These data confirm that malonyl-ACP is the high-affinity Mad substrate. However, the malonyl-CoA decarboxylase assay uses readily available, stable materials to assess Mad activity. MadA, MadA^C^, and MadB are malonyl-phosphopantetheine thioester decarboxylases, but MadA^N^ is not (Fig. 3*D*).

### Structure and mechanism of Mad

**Figure 4:**
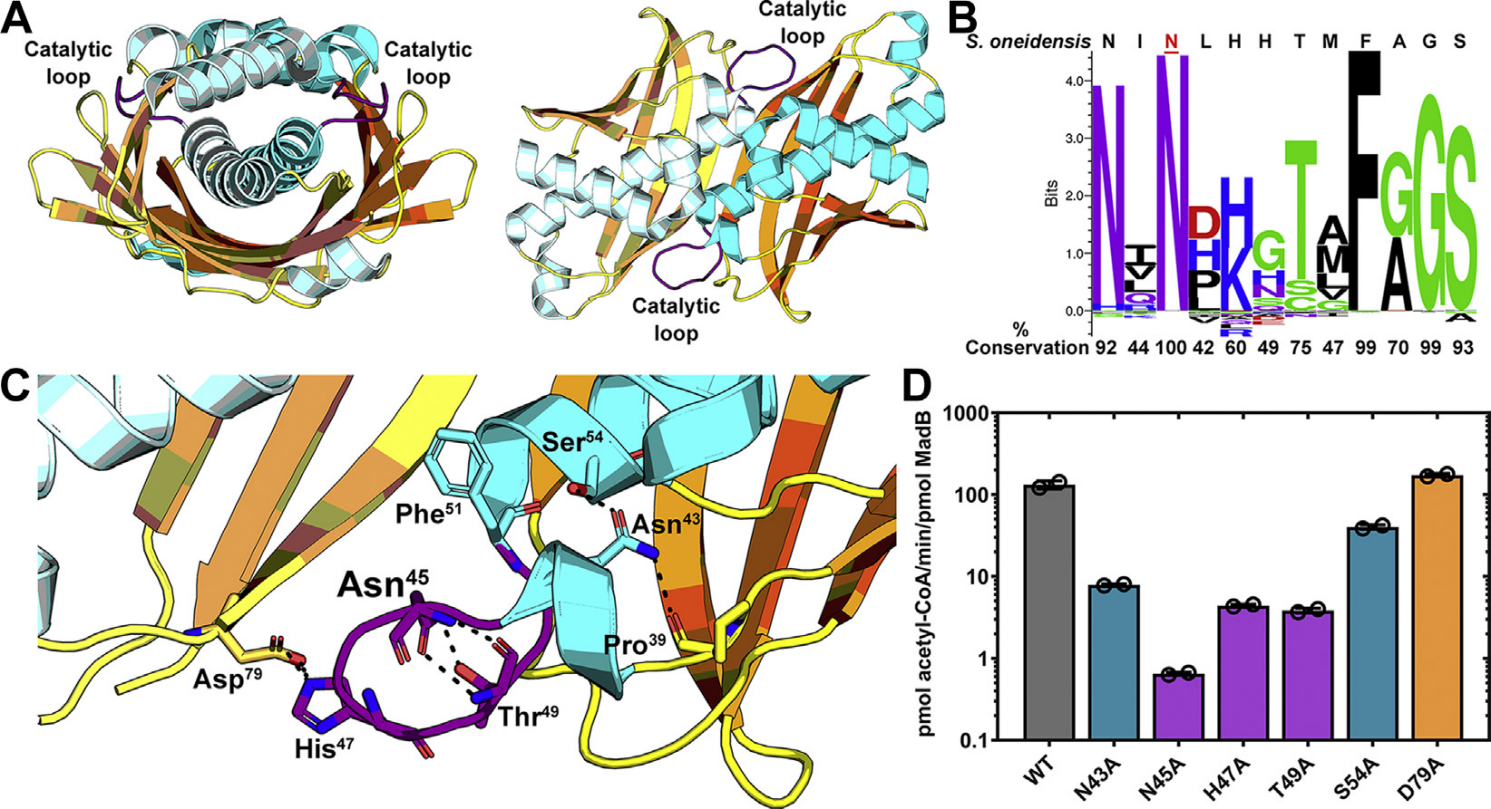
**Structure and catalytic loop of MadB.***A*, MadB (PDB ID: 1T82) has a hot dog fold architecture that is seen in two views of the protein. The location of the catalytic loop (*purple*) is labeled in both views. *B*, conserved residues within the loop sequence in the Mad protein family illustrating the high degree of conservation and the invariant Asn45. *C*, location of the mutated residues in the active site loop of MadB. Asp79 and His47 interact to position the loop, Thr49 and Asn45 form hydrogen bond interactions across the loop, and a Pro39-Asn43-Ser54 hydrogen bond network stabilize the base of the loop. *D*, catalytic activity of MadB mutants using the malonyl-CoA decarboxylase assay. Mad, malonyl-ACP decarboxylase.

Each protomer of *S. oneidensis* MadB has a prototypical hot dog-fold consisting of a central, hydrophobic, 6-turn α-helix (hot dog) surrounded by a six-stranded antiparallel β-sheet to create a dimer with two anti-parallel α-helices wrapped by 12 β-strands (Fig. 4*A*). The dimer interface is formed by the interaction between the α-helices and the β3 strand from each protomer. A prominent feature in the structure is the loop between residues 44 and 50 (Fig. 4*A*), and a bioinformatic analyses of Pfam_09500 sequences identifies this short segment as the most highly conserved patch of amino acids in the protein family (Fig. 4*B*). Asn45 and Phe51 are completely conserved in the Mad family, with Asn43 present in 97% of sequences. Phe51 has an obvious structural role in stabilizing the base of the loop by packing the phenyl ring into an adjacent hydrophobic pocket. Each of the loop residue side chains engage in interactions that stabilize the loop conformation, and in the case of Asn43, make direct contact with Asn45 (Fig. 4*C*). Site-directed mutagenesis of the MadB catalytic loop residues produced properly folded proteins based on their thermal stabilities and dimeric structures (Table S3), and each was analyzed for Mad activity using the malonyl-CoA decarboxylase assay (Fig. 4*D*). The MadB(S54A) and MadB(D79A) mutants had nearly normal malonyl-CoA decarboxylase activity. The decarboxylation activity decreased by 10-fold in the MadB(N43A), MadB(H47A), and MadB(T49A) mutants, illustrating that mutations in any of these conserved-loop residues compromise activity. The key catalytic residue was identified as Asn45 based on the reduction of decarboxylase activity by >100-fold in MadB(N45A) (Fig. 4*D*). These experiments identify the Ile44-Met50 loop as the Mad active site. The methylmalonyl-ACP decarboxylase encoded within the limoline polyketide gene cluster (LmnK) has a double hot dog fold and also uses an asparagine residue on a similar loop to catalyze decarboxylation (27, 28).

**Figure 5:**
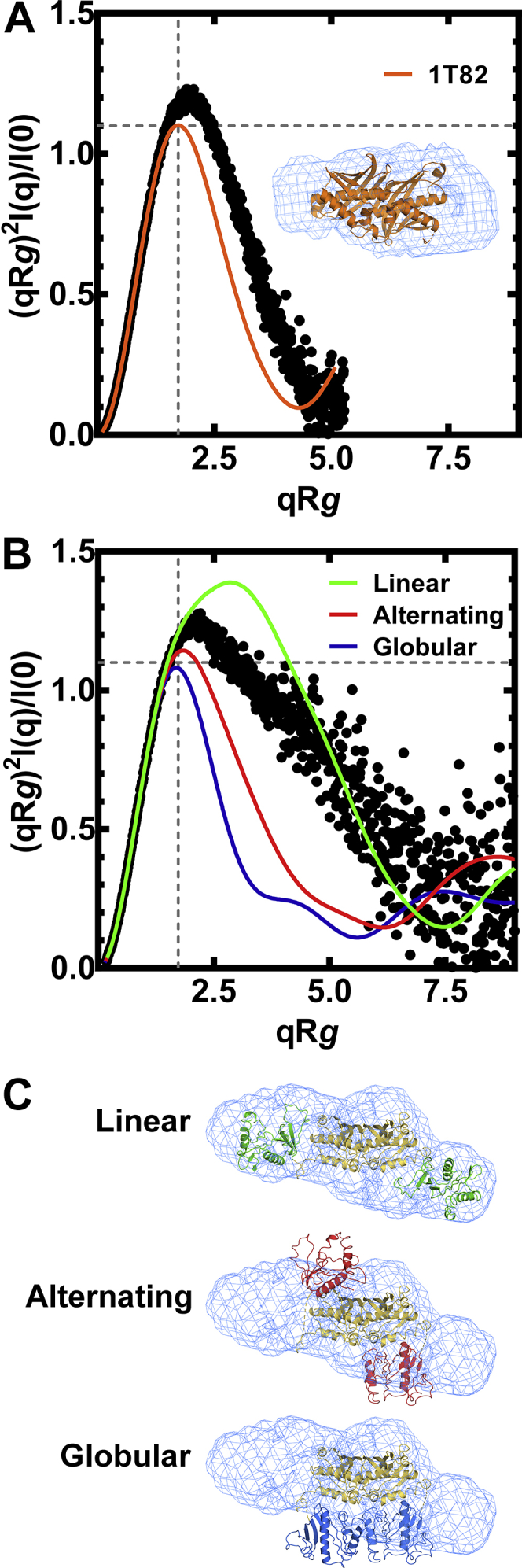
**Molecular architecture of MadA.** SEC-SAXS analysis was used to determine the molecular volumes occupied by MadA and MadB in solution. *A*, a normalized Kratky plot shows that MadB is best modeled as a globular protein in solution (χ^2^ = 9.44). *Inset*, MadB structure fit into the experimentally determined SAXS volume. *B*, a normalized Kratky plot showing that MadA does not behave as a globular protein. Three theoretical models for MadA were calculated assuming a linear (*green*), alternating (*red*), or globular (*blue*) arrangement of MadA^N^ and MadA^C^. The linear arrangement of MadA^N^ and MadA^C^ domains in solution best fit the experimental data (χ^2^ = 9.44), compared with alternating (χ^2^ = 15.3) or globular (χ^2^ = 37.2) conformations. *C*, three models for MadA architecture were visualized in the molecular volume of MadA calculated from SEC-SAXS data using PDB ID: 5JQ4 to model MadA^N^ and PDB ID: 1T82 to model MadA^C^. Mad, malonyl-ACP decarboxylase; SAXS, small angle X-ray scattering.

The architecture of the Mad proteins in solution was analyzed by size-exclusion chromatography-small angle X-ray scattering (SEC-SAXS) (29, 30, 31). MadB has a homogenous particle distribution, and the normalized Kratky plot is a close fit to a compact globular protein (Fig. 5*A*). Accordingly, the MadB crystal structure fits into the *ab initio* electron density map calculated from the SAXS data (32) (Fig. 5*A*, *inset*). MadA was also a homogenous particle population of dimers with a particle dimension of D_max_ = 139 Å and radius of gyration of R*g* = 38.21 ± 0.11 Å (Table S4). The normalized dimensionless Kratky plot shows a primary peak with a shoulder at low q that does not converge to the q axis (Fig. 5*B*). The bell-shaped profile with a peak occurring at qR*g* > √3 is consistent with a protein with ordered domains tethered together by a linker rather than being arranged in a globular conformation like MadB (Fig. 5*A*). Three theoretical models (globular, alternating, or linear) for the domain organization of MadA in solution were generated (33), and the linear model best fit the SAXS data set (Fig. 5*B*). Both the alternating and globular models show a prototypical peak at qR*g* = √3 (∼1.73) with a peak height of 3/*e* (∼1/1) (34) but were a very poor fit to the data (Fig. 5*B*). The differences between the linear model and the data at mid q suggest MadA samples a spectrum of compact and extended conformations (Fig. 5*B*). The *ab initio* electron density maps generated from the MadA solution scattering data (32) were superimposed on the globular, alternating, and linear MadA models to visualize the quality of the fits (Fig. 5*C*). The unmodeled electron density in the linear fit is attributed to the hydration shell and the independent movement of the MadA^N^ and MadA^C^ domains. We interpret these experiments to mean that the MadA^N^ domains are globular balls on chains connected to the hot dog MadA^C^ dimer.

### Complementation of ΔfabH strains

**Figure 6:**
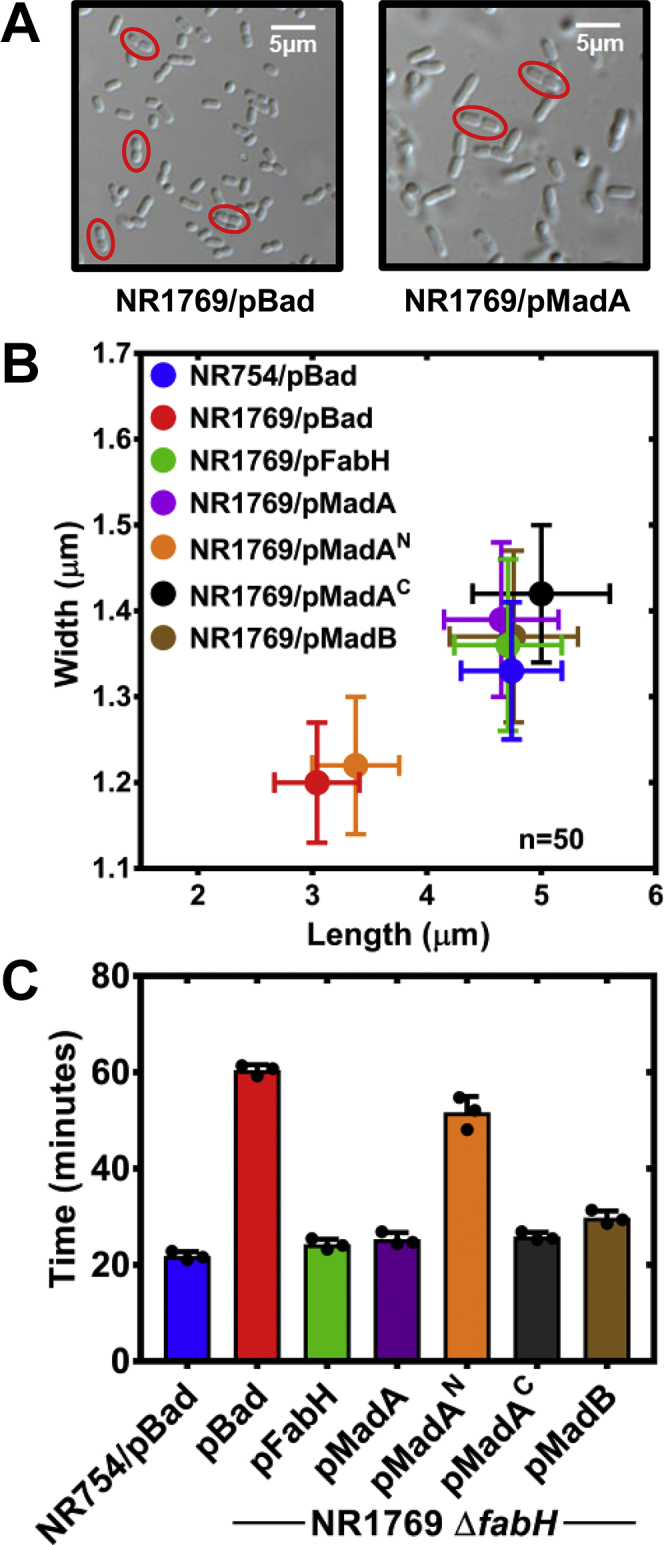
**Mad enzymes correct the growth and cell size defects in a Δ*fabH* strain.** A series of recombinant plasmids were prepared that expressed Mad proteins under control of the arabinose promoter. The strains were grown in LB with arabinose. *A*, micrographs of strain NR1769 (Δ*fabH*)/pBAD and NR1769 (Δ*fabH*)/pMadA illustrate the small cell phenotype of Δ*fabH* mutants and its complementation with MadA. The cells that met criteria for measurement are highlighted with *red circles*. *B*, the length and width of 50 cells with each genotype were measured, and the means ±SEM are plotted. *C,* doubling times were measured by regression analysis of the increase in A_600_ with time in three independent cultures. FabH, 3-ketoacyl-ACP synthase III; Mad, malonyl-ACP decarboxylase.

*E. coli* strain NR1769 (Δ*fabH*) lacks the major initiation enzyme and exhibits small cell and slow growth phenotypes (11, 12). A series of plasmids expressing different Mad proteins in the pBAD vector were used to assess the complementation of these phenotypes (Fig. 6). Immunoblotting showed that the His-tagged constructs were all expressed to approximately the same level (Fig. S4). The small cell phenotype was clearly visible in strain NR1769 (Δ*fabH*)/pBAD and was restored to normal in strain NR1769 (Δ*fabH*)/pMadA (Fig. 6*A*). This effect was quantified by selecting those cells within each field that possess an in-plane “figure 8” morphology for measurement (11) (Fig. 6*A*, red ovals). Cohorts of 50 cells were measured for each of the constructs and their lengths and widths plotted (Fig. 6*B*). The expression vectors harboring MadA, MadA^C^, and MadB were as effective as FabH in restoring cell size (both length and width) to the WT range. MadA^N^ expression did not restore cell size. In addition, the plasmids expressing either MadA, MadA^C^, or MadB were as effective as the FabH plasmid in correcting the doubling time defect in strain NR1769 (Δ*fabH*), but MadA^N^ expression did not (Fig. 6*C*). The amount of MadA protein in the cell is about an order of magnitude lower than FabH (35) suggesting why the endogenous expression level of *madA* is not sufficient to complement the Δ*fabH* phenotypes. Thus, elevated malonyl-ACP decarboxylase activity completely restores the growth rate and cell size defects caused by the absence of FabH.

### Acyl-CoA and acyl-ACP pools

**Figure 7:**
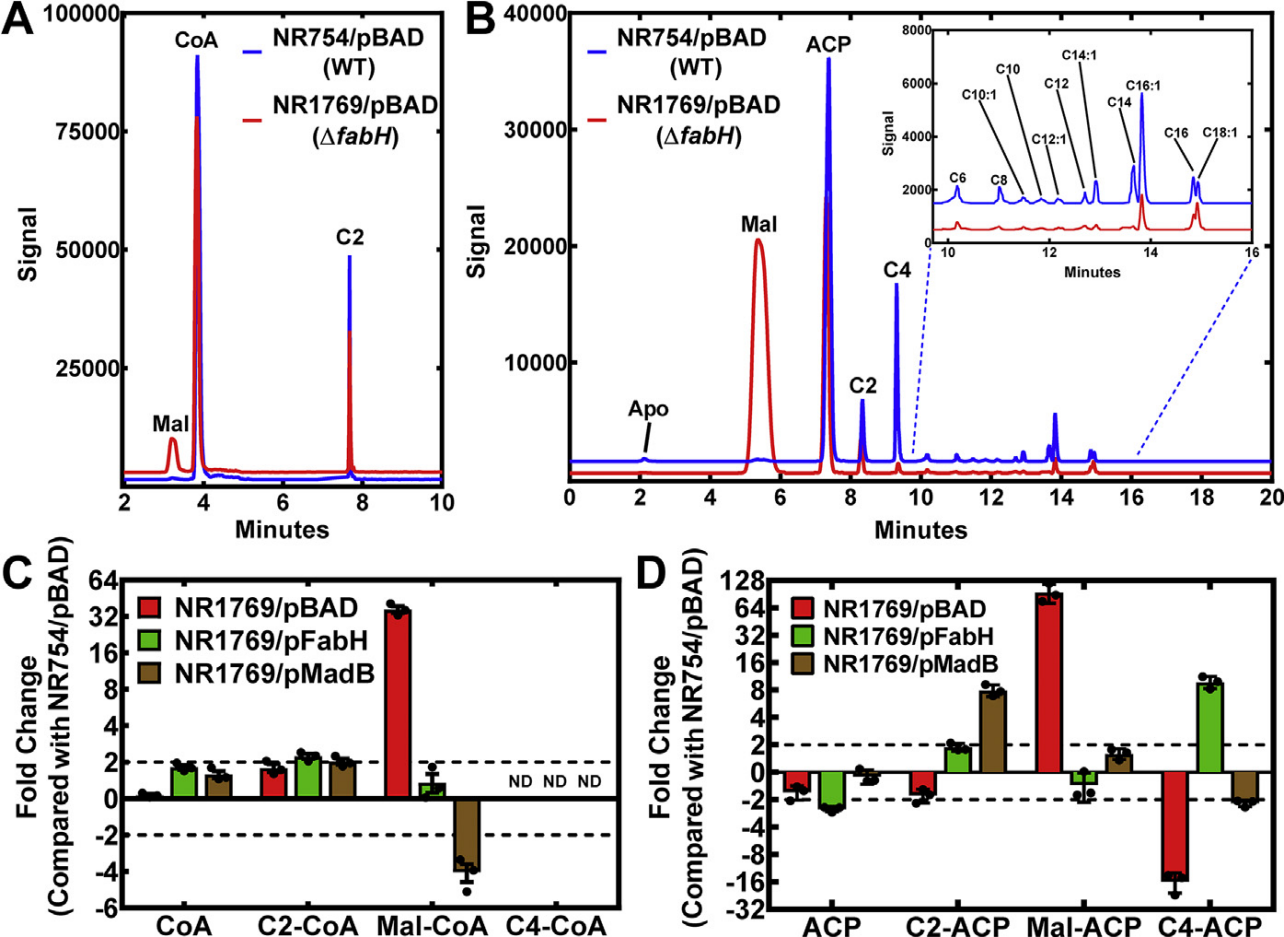
**Composition of the acyl-CoA and acyl-ACP intermediate pools in Δ*fabH* strains.***A*, representative experiment showing the primary data comparing the acyl-CoA pool composition in strain NR1769 (Δ*fabH*)/pBAD with its WT counterpart. *B*, representative experiments showing the primary data comparing the acyl-ACP pool composition in strain NR1769 (Δ*fabH*)/pBAD with its WT counterpart. *C*, fold change in the acyl-CoA pool in strains NR1769 (Δ*fabH*)/pBAD, NR1769 (Δ*fabH*)/pFabH, and NR1769 (Δ*fabH*)/pMadB compared with the WT strain NR754/pBAD. *D*, fold change in the short-chain acyl-ACP pool composition in strains NR1769 (Δ*fabH*)/pBAD, NR1769 (Δ*fabH*)/pFabH, and NR1769 (Δ*fabH*)/pMadB compared with the WT strain NR754/pBAD. ACP, acyl carrier protein; FabH, 3-ketoacyl-ACP synthase III.

The impact of FabH deletion and Mad complementation on the composition of the intracellular CoA and ACP thioester pools was examined *in vivo*. Acetyl-CoA is the major CoA thioester in WT cells, and there was an accumulation of malonyl-CoA coupled with a depletion of the nonesterified CoASH pool in strain NR1769 (Δ*fabH*) compared with WT (Fig. 7*A*). The major difference in the ACP thioester pool composition was also a large increase in malonyl-ACP in strain NR1769 (Δ*fabH*) compared with WT (Fig. 7*B*). The mass spectrometry data were normalized to either the [^13^C]acetyl-CoA (Fig. 7*C*) or [^13^C]acetyl-ACP (Fig. 7*D*) internal standards. The abundance of each intermediate was compared with the WT strain NR754. A 32-fold elevation in malonyl-CoA was the most notable feature in the Δ*fabH* cells (Fig. 7*C*). Complementation with FabH normalized the malonyl-CoA pool. Complementation with MadB also restored CoASH amounts, but there was a 4-fold decrease in the abundance of malonyl-CoA compared with WT. The relative abundance measurements showed that the Δ*fabH* deletion triggers a 64-fold increase in malonyl-ACP and a 16-fold decrease in butyryl-ACP, the only acyl-ACP intermediate dependent on FabH activity (Fig. 7*D*). Complementation with FabH normalized the malonyl-ACP abundance and also led to a substantial increase in butyryl-ACP. Complementation with MadB also normalized malonyl-ACP levels but led to a substantial increase in acetyl-ACP rather than butyryl-ACP (Fig. 7*D*). These measurements confirm the role of MadB as a malonyl-ACP decarboxylase *in vivo*. The Δ*fabH* mutation also led to substantially lower amounts of every acyl-, *trans*-2-enoyl-, and 3-hydroxyacyl-ACP intermediate in the cycle (Fig. S5). Complementation with FabH elevated these intermediates to higher levels than seen in WT cells showing that increased FabH initiation elevated FASII intermediates. MadB complementation restored the acyl-ACP intermediate levels to normal WT abundance. These data provide direct *in vivo* evidence that MadB is a malonyl-ACP decarboxylase and illustrate how Mad protein expression modifies the composition of the acyl-ACP pool.

### Acetyl-ACP is a GlmU substrate

**Figure 8:**
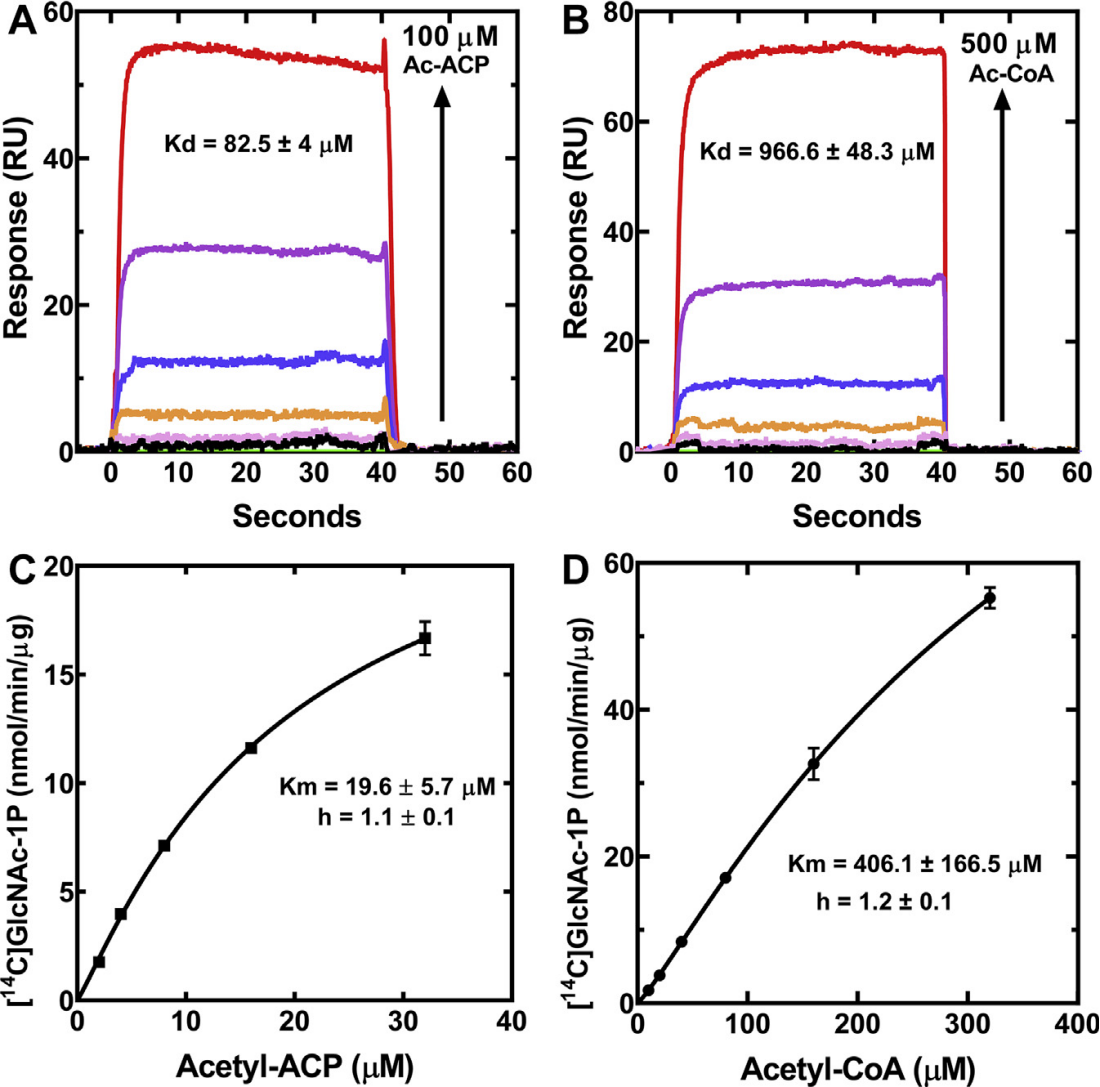
**GlmU has higher affinity for acetyl-ACP than for acetyl-CoA.** Surface plasmon resonance (SPR) was used to compare acetyl-CoA and acetyl-ACP binding with immobilized GlmU. *A*, SPR analysis of the binding of acetyl-ACP to GlmU. *B*, SPR analysis of acetyl-CoA binding to GlmU. The radiochemical TLC assay was used to determine the apparent K_M_s for acetyl-ACP and acetyl-CoA. The data were fit (*lines*) to a one-site binding Hill equation using GraphPad/Prism software. *C*, apparent K_M_ of GlmU for acetyl-ACP. *D*, apparent K_M_ of GlmU for acetyl-CoA. ACP, acyl carrier protein; GlmU, glucosamine-1-phosphate *N*-acetyl transferase/*N*-acetyl-glucosamine-1-phosphate uridylyltransferase.

GlmU is a bifunctional enzyme that converts glucosamine-1-phosphate to UDP-GlcNAc (36, 37, 38). UDP-GlcNAc is required for the initiation of LPS (LpxA) (39, 40), PG (MurA) (41, 42) and the ECA (WecA) (43). Because of its central position in cell-wall formation, GlmU is under development as an antibiotic target (44). GlmU, like MadA, was scored as an ACP-binding partner in a protein-protein interaction screen (45), suggesting that acetyl-ACP may be an *E. coli* GlmU substrate. GlmU has been characterized using acetyl-CoA as the acetyl donor and exhibits a relatively high K_M_ for acetyl-CoA (K_M_ = 320–650 μM) (36, 46, 47). We purified GlmU (Fig. S6*A*) and developed an assay (Fig. S6*B*) to determine if acetyl-ACP is a substrate. The binding of acetyl-ACP (Fig. 8*A*) and acetyl-CoA (Fig. 8*B*) to GlmU was assessed by surface plasmon resonance. These experiments showed GlmU-bound acetyl-ACP with 10-fold higher affinity than acetyl-CoA. GlmU is usually assayed by the spectrophotometric detection of the released CoA thiol (36, 46, 47) in 0.5 to 1 ml reactions, but a more sensitive assay is required to examine its affinity for a protein substrate. We developed an assay for the GlmU acetyltransferase half reaction in a 20 μl volume. The [^14^C]acetyl-ACP substrate and the *N*-[^14^C]acetylglucosamine-1-phosphate product were separated by thin-layer chromatography (Fig. S6*B*). The apparent K_M_ for [^14^C]acetyl-ACP was 20 μM (Fig. 8*C*) compared with 400 μM for acetyl-CoA (Fig. 8*D*). These data identify acetyl-ACP as a high affinity *E. coli* GlmU substrate.

## Discussion

**Figure 9:**
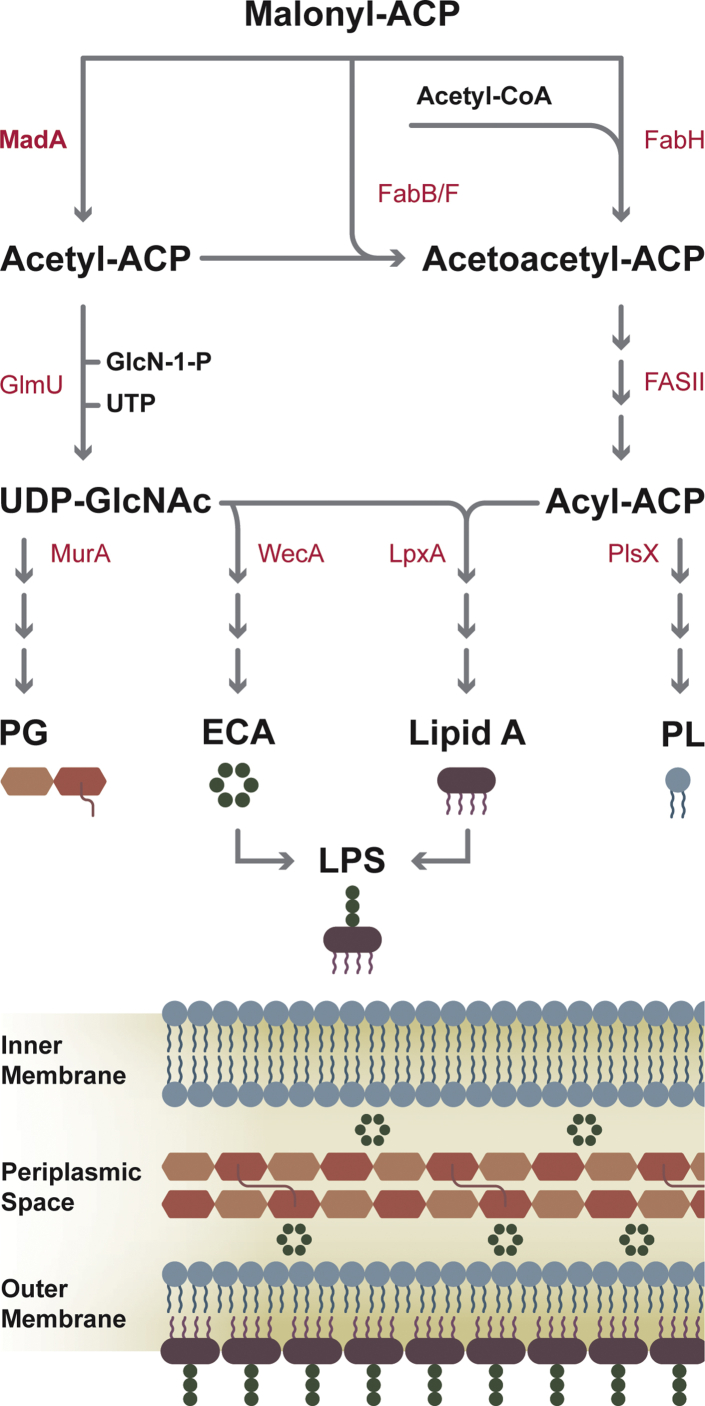
**Metabolic network involving MadA and acetyl-ACP.** Acetyl-ACP generated by malonyl-ACP decarboxylase (MadA) participates in the initial step in the biosynthesis of major elements that define the inner membrane, periplasmic space, and outer membrane architecture. Acetyl-ACP supports the initiation of fatty acid (FASII) and phospholipid (PL) synthesis by entering FASII at the FabB/F-condensing enzyme step. Acetyl-ACP also is a substrate for GlmU, a bifunctional glucosamine-1-phosphate N-acetyl transferase/N-acetylglucosamine-1-phosphate uridyltransferase producing UDP-GlcNAc. UDP-GlcNAc is used as a substrate to initiate lipopolysaccharide (LPS; LpxA), peptidoglycan (PG; MurA), and enterobacterial common antigen (ECA; WecA) biosynthesis. ACP, acyl carrier protein; Mad, malonyl-ACP decarboxylase.

This work identifies the members of Pfam_09500 as malonyl-ACP decarboxylases and define the role of MadA and acetyl-ACP in *E. coli* metabolism (Fig. 9). The Mad proteins are primarily expressed in Proteobacteria and come in two flavors: a GNAT-domain fused to a hot dog domain (MadA) or a standalone hot dog dimer (MadB). These enzymes produce acetyl-ACP that was first detected as a component of the ACP thioester pool decades ago based on metabolic labeling and electrophoretic analyses in *E. coli* (5, 9). More recent mass spectrometry methods confirm acetyl-ACP is a major component of the *E. coli* ACP pool (18, 19). The origin of the acetyl-ACP pool has been a mystery, and the discovery of MadA identifies an enzyme responsible for acetyl-ACP synthesis in Proteobacteria. FabH is the major initiation enzyme that condenses acetyl-CoA with malonyl-ACP to form acetoacetyl-ACP that enters the elongation cycle at the FabG step (3-ketoacyl-ACP reductase) (5, 6) (Fig. 9). Mad proteins bypass FabH by converting malonyl-ACP to acetyl-ACP that is used by FabB/F to form acetoacetyl-ACP (2, 3, 4), which like the FabH product, enters FASII at the FabG step (Fig. 9). *S. oneidensis* has four genes that encode FabH enzymes (48). FabH1 and FabH2 initiate straight- and branched-chain FASII, respectively, and the other two genes are dispensable (48). The quadruple knockout still grows, albeit at a much slower rate, illustrating the presence of yet another mechanism to initiate FASII. *S. oneidensis* MadB characterized in this study is a likely candidate for the FabH bypass pathway in this organism.

A second advance from this study is the identification of acetyl-ACP as a high affinity GlmU substrate (Fig. 8). GlmU is an essential enzyme that produces UDP-GlcNAc, a substrate required for the initiation of key cell-envelope components: LPS (LpxA) (39, 40), PG (MurA) (41, 42) and the ECA (WecA) (43) (Fig. 9). Because of its central importance in the synthesis of cell wall polysaccharides, GlmU has received considerable attention as an antibiotic drug-discovery target (44). Previous research has used acetyl-CoA as the GlmU substrate (36, 46, 47). Acetyl-CoA is indeed a substrate for GlmU, but our work shows that acetyl-ACP is a much higher affinity substrate for the enzyme. These data suggest that UDP-GlcNAc may be a major route for acetyl-ACP metabolism in *E. coli* (Fig. 9). It is not clear how widespread acetyl-ACP usage by GlmU is in the bacterial kingdom because the Mad family of proteins are restricted to the Proteobacteria. GlmU is a key enzyme in cell wall biogenesis in all bacteria, which either indicates that acetyl-ACP does not support GlmU activity in most bacteria or that a different gene family encodes a malonyl-ACP decarboxylase in these bacteria. Thus, the Mad proteins supply acetyl-ACP for the initiation of the biosynthetic pathways for four major cell envelope components in Proteobacteria (Fig. 9).

The discovery of a family of malonyl-ACP decarboxylases identifies a new player in cell envelope biosynthesis in Proteobacteria, but many questions remain to be addressed. MadA is not an essential enzyme, therefore more experimental work will be required to understand the physiological setting(s) where Mad dependent acetyl-ACP formation is beneficial or of regulatory significance. The intracellular levels of acetyl-CoA vary by an order of magnitude on different carbon sources (49, 50), suggesting that MadA may play a more important role in nutrient-limiting conditions. It is possible that acetyl-ACP may be a substrate in other unidentified reactions. Mad proteins appear confined to Proteobacteria, suggesting that acetyl-ACP formation may be most important in supporting fatty acid synthesis for LPS, an essential outer membrane constituent confined to this group of bacteria. The role of the amino terminal domain of the MadA remains to be elucidated. It is not required for malonyl decarboxylase activity and is not present in the majority of *Mad* genes in Proteobacteria. The MadA^N^ configuration is confined to γ-Proteobacterial species, suggesting it has a specific function in these organisms. MadA^N^ and MadA^C^ are clearly independently folded protein domains opening the possibility that standalone GNAT enzymes carry out the MadA^N^ function in Proteobacteria that express MadB. There are many candidates for a MadA^N^-like protein among the GCN5-related *N*-acetyl transferases of unknown function that are present in all bacterial genomes. We have not documented any impact of the MadA^N^ domain on MadA^C^ decarboxylase activity so perhaps MadA^N^ carries out the acetylation of lysine residues on an unidentified target protein, as suggested by its homology to known GNAT *N*-acetyl transferases.

## Experimental procedures

### Materials

Malonyl CoA, NADPH, acetyl-CoA, magnesium chloride, sodium chloride, SYPRO orange gel stain, thrombin, glucoseamine-1-phosphate, and monoclonal anti-His_6_ alkaline phosphatase conjugated antibody were purchased from MilliporeSigma. [1-^14^C]Acetyl-CoA (specific activity 60 mCi/mmol) and [2-^14^C]malonyl CoA (specific activity 55 mCi/mmol) were purchased from PerkinElmer and American Radiolabeled Chemicals, respectively. [U-^13^C]acetyl-CoA was from MilliporeSigma. Thrombin (Sigma T4648), streptavidin agarose (Thermo 20359), HPDP-biotin (Thermo 21341), and glucoseamine-1 phosphate (Sigma G9753) are used in this study. The bacterial media was purchased from BD Medical Technologies. Antibiotics, nickel resin, and DTT were purchased from GoldBio. Protease inhibitor and Cytiva Amersham ECF Substrate for Western Blotting were purchased from Thermo Fisher. Bacterial strains (Table S5) and plasmids (Table S6) are used in this study.

### Protein purification

The YiiD (MadA) sequence from *E. coli* (Uniprot ID: P0ADQ2) and the domains MadA^N^ and MadA^C^ were amplified from Top10 genomic DNA and cloned by Gibson Assembly into pET28a (cut with NdeI + HindIII) under control of an IPTG inducible promotor to create plasmids pPJ604, pPJ605, and pPJ606. The sequence for MadA^N^ included amino acids 1 to 161, and the sequence for MadA^C^ included amino acids 171 to 329. A PCR product of the *E. coli GlmU* gene was cloned into NdeI-BamHI site of pET28a using Gibson Assembly to obtain pPJ602 and purified, as described (47). The DNA sequence of *MadB* (Uniprot ID: Q8E989) was cloned into the pET28a expression vector under control of an IPTG inducible promotor to create plasmid pPJ612. The expression vectors for mutant MadB proteins were constructed by site-directed mutagenesis using the QuikChange Lightning kit.

All the proteins were expressed using pET expression vectors in *E. coli* BL21(DE3) cells. The constructs for ACP, FabH, FabD, and FabG have been described previously (13). Protein expression was induced with IPTG for 3 h at 37 °C (ACP, FabH, FabD, FabG, and GlmU) or overnight at 16 °C (MadA, MadA^N^, MadA^C^, MadB, and MadB mutants). After cell lysis, the protein was purified using nickel affinity chromatography. The pooled purified protein was either dialyzed overnight (ACP, FabH, FabD, FabG, and GlmU) or further purified by size-exclusion chromatography (MadA, MadA^N^, MadA^C^, MadB, and MadB mutants). The His_6_-tag was then cleaved from ACP and MadA^C^ by thrombin digestion and separated from the purified proteins by nickel affinity chromatography. GlmU was expressed in BL21(DE3) for 3 h at 37 °C after induction with 1 mM IPTG. The protein was purified, as described (47). His-tagged GlmU was dialyzed overnight in 20 mM Tris, pH 7.5, 300 mM NaCl, 0.1% BME, 10% glycerol, and 1 mM EDTA. The proteins were concentrated using Amicon Ultra centrifugal filters and quantified by UV absorption with the exception of ACP, which was quantified by the BCA protein assay kit (Pierce).

The protein expressed from the *MadA* gene resulted in two bands on SDS gel electrophoresis. The second band was slightly lower and less intense than the band running at the expected location, indicating a second start site in the construct. The sequence analysis revealed a methionine at position 17 of the annotated protein sequence that had an apparent ribosome-binding site upstream. Therefore, we used site directed mutagenesis of plasmid pPJ604 (QuikChange Lightning kit; Agilent) to create the MadA(M17A) mutant that purified as a homogenous protein. The MadA(M17A) mutant had the same thermal stability and enzyme activities as MadA. The experiments were performed using the MadA(M17A) construct.

### Enzyme assays

Condensing enzyme activity of FabH and MadA was assayed in a reaction mixture containing: 0.1 M Tris, pH 7, 0.2 M NaCl, 30 μM ACP, 0.1 mM NADPH, 0.05 μg FabD with or without 0.5 μM FabG. In reactions following the acetyl-CoA carbon, 35 μM malonyl-CoA and 30 μM [^14^C]acetyl-CoA were added, and in reactions following the malonyl-CoA carbon, 30 μM acetyl-CoA and 35 μM [^14^C]malonyl-CoA were added. The ACP was reduced with 0.25 mM DTT before the other reaction components were added. The reactions were initiated by the addition of 1 nM FabH or 5 nM MadA. After incubation at 37 °C for 12 min, the reaction mixtures were placed on ice. The acyl-ACP was then separated on a conformationally sensitive 4 to 20% gradient polyacrylamide gel containing 0.5 M urea, dried and imaged with a Typhoon PhosphoImager. Malonyl-ACP decarboxylase activity of MadA, MadA^N^, MadA^C^, and MadB was assayed in a reaction mixtures containing 0.1 M Tris, pH 7, 0.2 M NaCl, and 30 μM [^14^C]malonyl-ACP. The reactions were initiated by the addition of Mad followed by incubation at 37 °C for 12 min. The products were analyzed by separation on a conformationally sensitive 13% polyacrylamide gel containing 0.5 M urea.

Mass spectrometry was used to confirm that acetyl-ACP is the product of the MadA reaction. The reaction mix contained 0.1 M Tris-HCl, pH 7, 0.2 M NaCl, 30 μM ACP, 0.6 μg FabD, and 35 μM malonyl-CoA. ACP was reduced with 0.25 mM DTT before the other reaction components were added. The reaction was initiated by the addition of 50 nM MadA. An acetyl-ACP standard in 0.1 M Tris-HCl, pH 7, plus the reaction mix without the addition of MadA were used as the control. After incubation at 37 °C for 12 min, the reaction was stopped on ice. The ACP samples were digested with Asp-N (Sigma Millipore), as described below. Acetyl-ACP was detected by LC-MS/MS using the *m/z* = 716.3/303.2 (Q1/Q3) masses characteristic for acetyl-ACP.

The reactions used to confirm that *E. coli* FabB and *S. pneumoniae* FabF condensed malonyl with acetyl-ACP contained 0.1 M Tris-HCl, pH 7, 0.25 mM DTT, 100 μM ACP, 0.2 M NaCl, 0.5 ng FabD, 0.1 mM NADPH, 10 nM FabG, 100 μM malonyl-CoA, and 50 μM acetyl-ACP. The reactions were initiated with 1 μM FabB or 1 μM FabF. FabG was included in the reaction to convert the 3-ketobutyryl-ACP product to the more stable 3-hydroxybutyryl-ACP. After incubation at 37 °C for 1 h, the ACP was digested with Asp-N protease, as described below. The 4-hydroxyutyryl-ACP product was identified by LC-MS/MS using the *m/z* = 760.3/347.2 (Q1/Q3) masses.

Malonyl-CoA decarboxylase activity of MadA was measured in the reaction mixtures containing: 0.1 M Tris, pH 7, 0.2 M NaCl, and 4 mM malonyl-CoA. The reactions were initiated by protein addition and incubated for 12 min at 37 °C. An equal volume of ice-cold methanol was added to stop the reactions, and the samples were centrifuged to remove any precipitated protein. CoA thioesters were quantified using a liquid chromatography (HPLC) method, as previously described (51).

### Synthesis of labeled acyl-ACP

^13^C- and ^14^C-labeled acyl-ACPs were synthesized by the previously described method for crotonyl-ACP synthesis (26). The [U-^13^C]acetyl-ACP reaction mixture contained the following: 300 μM purified ACP with 2 μM *E. coli* ACP synthase and 600 μM [U-^13^C]acetyl CoA in 0.05 M Tris, pH 7, with 0.01 M MgCl_2_. The [1-^14^C]acetyl-ACP reaction contained the following: 300 μM purified ACP with 4 μM *E. coli* ACP synthase, 300 μM acetyl-CoA, and 150 μM [1-^14^C]acetyl-CoA in 0.1 M NaPO_4_, pH 7, with 0.01 M MgCl_2_. The [2-^14^C]malonyl-ACP reaction contained the following: 300 μM purified ACP with 4 μM *E. coli* [ACP]synthase, 800 μM malonyl-CoA, and 400 μM [2-^14^C]malonyl-CoA in 0.1 M Tris, pH 8.5, with 0.01 M MgCl_2_. All the reactions were incubated for 2 h at 37 °C. The His_6_-tagged [ACP]synthase was removed from the reactions by nickel affinity chromatography. The MgCl_2_ and excess CoA were removed using a PD-10 desalting column. The labeled acyl-ACPs was eluted in 0.02 M Bis-Tris, pH 6, with 0.2 M NaCl and concentrated using Amicon Ultra centrifugal filters (3 kDa cut-off). The concentrated standards were quantified using the BCA protein assay kit (Pierce).

### Thermal stability assays

Thermal stability of purified proteins was determined by monitoring protein binding to a fluorescent dye with increasing temperature using an Applied Biosystems 7500 Fast Real Time PCR System according to previously published protocols (52, 53). The samples containing 10 μM protein in 0.05 M NaPO_4_, pH 7, 0.3 M NaCl, and 2.5× SYPRO Orange dye were loaded into 96 well ThermoGrid plates (Denville Scientific) and sealed. The plates were centrifuged at 1000*g* for 5 min. The samples were heated from 25 °C to 95 °C at 1 °C/min with a fluorescence measurement taken at each degree increase using a TAMRA filter set (Ex 560 nm and Em 582 nm). The resulting data was fit to the first derivative of the Boltzman sigmoidal equation using GraphPad/Prism software to determine the point at which 50% of the protein was denatured. The experiments were completed in triplicate.

Thermal stability of the MadB mutants was also determined by nanoDSF. Dynamic light scattering of the proteins was measured with the Prometheus NT.48 (NanoTemper Technologies). Briefly, 20 μl samples containing 20 mM Tris, pH 7.5, 0.2 M NaCl, 10 mM EDTA, and 10 μM MadB mutant protein were mixed and loaded into Prometheus NT.48 Series nanoDSF Grade High Sensitivity Capillaries (NanoTemper Technologies) by capillary force action. The capillaries were heated from 20 °C to 90 °C at a rate of 1 °C/min, and the UV light scattering was recorded to determine the Tagg.

### Analytical ultracentrifugation

Sedimentation velocity experiments were conducted in a ProteomeLab XL-I analytical ultracentrifuge (Beckman Coulter) after standard protocols unless mentioned otherwise (54). The samples in buffer containing 0.02 M Tris, pH 7.5, and 0.5 M NaCl were loaded into cell assemblies comprised of double sector charcoal-filled centerpieces with a 12 mm path length and sapphire windows. The buffer density and viscosity were calculated using the software SEDNTERP (http://www.jphilo.mailway.com/download.htm) (55). The partial specific volumes and the molecular masses of the proteins were calculated based on their amino acid compositions in SEDFIT (https://sedfitsedphat.nibib.nih.gov/software/default.aspx). The cell assemblies, containing identical sample and reference buffer volumes of 390 μl, were placed in a rotor and temperature equilibrated at rest at 20 °C for 2 h before it was accelerated from 0 to 50,000 rpm. Rayleigh interference optical data were collected at 1-min intervals for 12 h. The velocity data were modeled with diffusion-deconvoluted sedimentation coefficient distributions in SEDFIT (https://sedfitsedphat.nibib.nih.gov/software/default.aspx), using algebraic noise decomposition and with signal-average frictional ratio and meniscus position refined with nonlinear regression (56). The s-values were corrected for time, and finite acceleration of the rotor was accounted for in the evaluation of Lamm equation solutions (57). Maximum entropy regularization was applied at a confidence level of P-0.70.

### Homology modeling

MadA homology models were generated using SWISS-MODEL (58), using the *S. aureus* putative acetyltransferase SACOL1063 (PDB: 5JQ4) and *S. oneidensis* thioesterase (PDB: 1T82) structures as templates for the amino (19–162) and carboxy (171–314) terminal domains, respectively. The theoretical MadA conformer domain assemblies were arranged in PyMOL (59).

### Phylogeny

All sequences containing the Mad domain (PF_09500) in the Pfam protein database were aligned using MAFFT (60). A logo for the active site loop was created from this alignment using the online Seq2Logo tool (61).

### Complementation strain construction

For the *in vivo* complementation experiments, FabH and Mad proteins were expressed in strain NR1769 (Δ*fabH*). The sequences encoding His-tagged versions of FabH, MadA, MadA^N^, MadA^C^, and MadB were inserted into the pBAD vector (Thermo Fisher), which contains an arabinose inducible promoter. The plasmids were transformed into strain NR1769 by electroporation, and the positive transformants were selected by plating on kanamycin and carbenicillin.

Protein expression of complementation strains was confirmed by Western blotting. The overnight cultures were cut back to an optical density of 0.05 in LB and grown for 2 h at 37 °C with shaking. A_600_ were adjusted to 0.05 in Luria broth with 0.1% arabinose to induce expression of His_6_-tagged proteins from pBAD vectors. The cultures were incubated 75 min at 37 °C with shaking. The cell pellets were collected by centrifugation and stored at −80 °C. The pellets were resuspended in 0.02 M Tris, pH 7.9, 0.5 M NaCl, 10% glycerol, and 0.01 M imidazole with protease inhibitor and lysed in a French press. The samples were centrifuged at 20,000*g* for 30 min at 4 °C to remove insoluble debris, and the protein was quantified using BCA assay and equal amounts of protein were separated on a 10% Bis-Tris gel. The protein was transferred to a polyvinylidene difluoride membrane using the iBlot 2 Dry Blotting System (Thermo Fisher). The membrane was incubated in 5% milk for 1 h at RT, then overnight at 4 °C in a 1:2000 dilution of anti-His_6_ alkaline phosphatase-conjugated monoclonal antibody in 5% milk. The membrane was washed in Tris buffered saline with 0.1% Triton X-100 for 15 min intervals for a total of 1 h. ECF substrate was added for 1 to 3 min, and the membrane was imaged using the Typhoon 9200 PhosphoImager.

### Doubling time and cell size

Doubling time and cell size estimation experiments were performed according to previously published methods with modifications (11, 12). The overnight cultures were diluted to an A_600_ of 0.05 in LB and allowed to incubate at 37 °C for 2 h. The cultures were diluted to 0.05 again, and 0.1% arabinose was added. The cultures were incubated at 37 °C for 6 h with aliquots removed at 20 min intervals to record the A_600_. The doubling times were determined by plotting A_600_ *versus* time in GraphPad/Prism and fitting the linear portion of the curve to the exponential growth equation. The experiments were performed in triplicate.

The samples for cell size estimation were removed from the above growth experiments when cultures reached an A_600_ of 0.2 to 0.4. One ml of the culture was centrifuged at 13,000 rpm for 1 min. The supernatant was removed, and the cells were resuspended in 1 ml 4% formaldehyde in phosphate buffered saline, pH 7. The cells were incubated for 15 min at RT followed by 15 min on ice and then washed twice with PBS. The cells were then resuspended in a final volume of 25 μl PBS. Two μl of the cells was spotted onto a coverslip and allowed to dry. The coverslips were mounted with 15 μl 50% glycerol in PBS and allowed to dry for at least 1 day. Differential Interference Contrast imaging was performed using a 100× objective on a Nikon Eclipse Ni Widefield Microscope. The length and width measurements of 50 dividing cells with a figure “8” morphology for each strain were recorded using Nikon NIS-Elements imaging software.

### Acyl-CoA quantification

Overnight cultures of strains NR1769/pBAD, NR1769/pFabH, and NR1769/pMadB were diluted to A_600_ = 0.05 in M9 medium with 1 mM MgSO_4_, 0.1 mM CaCl_2_, and 0.4% glucose. After 2 h, the incubation at 37 °C cultures were diluted to A_600_ = 0.05 in the same media and supplemented with 0.1% arabinose to induce expression of FabH or MadB. The cultures were incubated at 37 °C until they reached an A_600_ = 0.6. The extraction method, liquid chromatography column and gradient, mass spectrometry parameters, and the Q1 and Q3 masses for the acyl-CoA were exactly as described (17).

### Acyl-ACP quantification

The samples were prepared for acyl-ACP quantification, as previously described (17, 18). One milliliter aliquots of cultures at mid-log phase growth were removed to a 1.5 ml Eppendorf tube containing 250 μl ice cold 10% trichloroacetic acid. The tubes were inverted and centrifuged to collect the precipitate that was washed with acetone, dried, and stored at −80 °C. The pellets were resuspended in 100 μl of lysis buffer (0.05 M NaPO_4_, pH 7.2, 1 mM ascorbic acid, 2 mM ethylenediaminetetraacetic acid, and 6 M urea) prepared just before use. [^13^C_2_]Acetyl-ACP was added as an internal standard (1 μl of 0.0125 μg/μl [U-^13^C]acetyl-ACP). A chloroform/methanol extraction was performed by adding 400 μl methanol, followed by 100 μl of chloroform. The samples were sonicated in a sonication bath (Fisher Scientific CPXH Series 1.9L) for 10 min at room temperature to resuspend. The phases were separated by adding 300 μl of 200 mM formate buffer (pH 3.9). The upper phase was discarded, and 300 μl methanol was added to the remaining sample. The precipitated proteins were washed with 300 μl of methanol and then dried. ACP was resuspended in 10 μl of 0.1 M NaPO_4_, pH 6.5, and sonicated for 10 min at room temperature in an ultrasonic bath. Insoluble debris was pelleted, and 5 μl of resuspended ACP was removed to a clean tube. ACP was digested by adding 0.5 μg endoproteinase Asp-N (MilliporeSigma) in 10 μl of 0.1 M Tris, pH 7.5 (62). The samples were incubated 1 h at room temperature to allow for complete digestion with minimal degradation of acyl chains. Proteolysis was quenched with 15 μl methanol. The liquid chromatography column and solvent gradient, mass spectrometry parameters, and Q1 and Q3 masses for acyl-ACPs were exactly as described (17).

### GlmU assay

GlmU assays were performed with [1-^14^C]acetyl-CoA and [1-^14^C]acetyl-ACP. ACP in pET15b was expressed in BL21(DE3) and purified using Ni^2+^-NTA affinity chromatography. The His-tag from the His_6_-ACP was cleaved using thrombin overnight at room temperature, and the reaction was precipitated using 1% TCA. The pellet was resuspended in 20 mM Bis-Tris, pH 6.5 and centrifuged to remove any precipitated protein that did not go into solution. A Zeba spin column (Thermo Fisher) was used to exchange the buffer to 20 mM Tris, pH 7.5. DTT was added to a final concentration of 10 mM and incubated at 37 °C for 1 h to reduce the contaminating ACP; the excess DTT was removed using a Zeba spin column, and the buffer was changed to PBS containing 1 mM EDTA and 250 mM NaCl. To remove ACP, the eluate was mixed with HPDP-Biotin for 2 h at room temperature, and a Zeba spin column was used to remove any unreactive HPDP-Biotin. Streptavidin agarose was used to remove ACP bound to HPDP-Biotin; the supernatant contained pure apo-ACP was then heated at 95 °C for 10 min to destroy contaminating ACP-binding proteins. This step was essential to remove GlmU (and other FASII proteins) from the preparation. [1-^14^C]Acetyl-CoA (specific activity 60 mCi/mmol) was used to generate [^14^C]acetyl-ACP using [ACP]synthase, briefly the reaction mixture containing 100 mM Tris, pH 7.0, 10 mM MgCl_2_, 300 μM apo-ACP, 400 μM [1-^14^C]acetyl-CoA, and 4 μM [ACP]synthase was incubated at 37 °C for 2 h. Ni^2+^-NTA was added to remove the His-tagged [ACP]synthase, and the buffer was exchanged using a PD-10 column to 20 mM Bis-Tris, pH 6.0, and 200 mM NaCl. Radioactive GlmU assay contained the following: 20 mM KH_2_PO_4_, 20 mM MgCl_2_, 0.1% BME, 500 μM glucoseamine-1-phosphate (GlcN-1P), [^14^C]acetyl ACP (0–32 μM), and 0.6 ng of GlmU. The reaction was incubated for 15 min at 37 °C, and 10 μl of the reaction was spotted on a Silica Gel H plate and developed with 1-butanol:methanol:ammonia:water (5:4:2:1, v/v/v/v). The reaction with [^14^C]acetyl-CoA (0–320 μM) (specific activity 20 mCi/mmol) was performed similarly but with 3 ng of GlmU. The bands on the plate were quantified using a Typhoon 9200 PhosphoImager and ImageQuant. The experiment was repeated three times, and the data were fit to one site binding Hill equation (GraphPad/Prism software).

### Surface plasmon resonance

The experiments were conducted at 20 °C using a Pioneer optical biosensor (Sartorius). His-tagged *E. coli* GlmU was immobilized on a polycarboxylate hydrogel-coated gold chip preimmobilized with nitrilotriacetic acid (His Cap chip; Sartorius) by capture-coupling, a hybrid method of capture and amine coupling chemistry (63). The chip was primed in chelating buffer (10 mM Hepes, pH 7.4, 150 mM NaCl, 50 μM EDTA, and 0.005% Tween 20) and was preconditioned at 10 μl/min with three 60 s injections of wash buffer (10 mM Hepes, pH 8.3, 150 mM NaCl, 350 mM EDTA, and 0.05% Tween-20) and one 60 s injection of chelating buffer before being charged with a 60 s injection of 500 μM NiCl_2_ in chelating buffer. The charged chip was primed with immobilization buffer (10 mM Hepes pH 7.5, 300 mM NaCl, 1 mM TCEP, 10% glycerol, and 0.005% Tween 20), and carboxyl groups on the hydrogel were activated with *N*-ethyl-*N′*-(3-dimethylaminopropyl) carbodiimide and *N*-hydroxysuccinimide. GlmU was injected over the activated surface to immobilization levels of ∼750 RU for acetyl-ACP binding and ∼2000 RU for acetyl-CoA binding. One channel on the chip was charged with Ni^2+^ and activated with EDC/NHS without adding protein to be used as a reference cell. Blocking of any remaining active sites was achieved by priming with binding buffer containing Tris (20 mM Tris-HCl, pH 7.5, 300 mM NaCl, 1 mM TCEP, 10% glycerol, and 0.005% Tween 20). Acetyl-CoA or acetyl-ACP was prepared in binding buffer as a three-fold dilution series with maximum concentration of 500 μM or 100 μM, respectively, and was injected in triplicate for each concentration at a flow rate of 75 μl/min. A series of buffer-only (blank) injections was included to account for instrument noise. The data were processed, double-referenced, and analyzed using the software package Qdat (version 4.3.1.2, Sartorius). The equilibrium dissociation constants were determined by equilibrium affinity analysis using a 1:1 (Langmuir) binding model.

### Malonyl-CoA and malonyl-ACP stability

Stabilities of malonyl-ACP and malonyl-CoA were determined by incubating 10 μg malonyl-ACP or 4 mM malonyl-CoA in 0.02 M Bis-Tris, pH 6, and 0.2 M NaCl for 1 or 16 h at room temperature, 30 °C or 37 °C. After incubation, the malonyl-ACP samples were separated using a conformationally sensitive urea gel (0.5 M urea, 13% acrylamide), stained with Coomassie dye and imaged using the Bio-Rad Gel Doc XR+ imaging system. After incubation, 40 μl of the methanol was added to 10 μl of each malonyl-CoA condition, and the CoA was quantified using the HPLC method previously described (51).

### SEC-SAXS

SEC-SAXS experiments were performed at the BioCAT beamline 18-ID-D at the Advanced Photon Source. Photons that scattered from the λ = 1.033 Å X-ray beam were recorded on the Pilatus3 X 1M detector at a sample-to-detector distance of 3.631 m, accessing a range of momentum transfer (q) from 0.0047 to 0.35 Å^−1^. A Superdex 200 10/300 increase column was preequilibrated with 20 mM Tris, pH 7.5, 200 mM NaCl, 1% glycerol, and 10 mM EDTA. MadA (290 μl, 6.1 mg/ml) was injected onto the column with a flow rate of 0.6 ml/min that enabled the separation of potential aggregates before flowing MadA through a temperature-controlled quartz capillary (1.0 mm internal diameter) flow cell for X-ray exposure. SAXS was performed in-line with this size-exclusion chromatography setup. One peak of X-ray scattering was detected by 0.5 s SAXS exposures of fractions of the column recorded every second. Buffer background was obtained from a baseline region of the chromatogram and subtracted from the averaged central portion of the MadA peak to obtain the scattering profile. The data were reduced, processed, and the overall parameters computed following standard procedures of the software package RAW (64) version 2.0.3. A linear Guinier plot indicated there was no significant radiation damage during the exposure period (not shown). The zero-angle intensity I_0_, radius of gyration R*g*, and associated uncertainties for these parameters were obtained by weighted linear regression of log(I) *versus* q^2^ as shown in the Guinier plot. The particle dimension D_max_ was determined from the pair distance distribution function P(r) with the program GNOM (65, 66). Twenty reconstructions were aligned, and all were averaged and refined in slow mode using DENSS (32) to calculate the electron density for the final 3-D reconstruction.

## Data availability

All data are presented in the article.

## Supporting information

This article contains supporting information.

## Conflict of interest

The authors declare that they have no conflicts of interest with the contents of this article.
