# Supplementary material for: Malonyl-acyl carrier protein decarboxylase activity promotes fatty acid and cell envelope biosynthesis in Proteobacteria
Source: J Biol Chem. 2021 Nov 18;297(6):101434. doi: 10.1016/j.jbc.2021.101434 (PMC8666670; doi:10.1016/j.jbc.2021.101434)
Supplement: Supplementary file 1 — Tables S1–S6 and Figures S1–S6 [file mmc1.pdf]

Supporting Information

Malonyl-acyl carrier protein decarboxylase supports fatty acid and cell envelope biosynthesis in Proteobacteria

Sarah G. Whaley, Christopher D. Radka, Chitra Subramanian, Matthew W. Frank, and Charles O. Rock[1]
From the Department of Infectious Diseases, St. Jude Children's Research Hospital, Memphis, Tennessee 38105

Table S1. Summary of results from sedimentation velocity experiments

**Sample**	s_{20}[a]	$s_{20,w}$[b]	***MW***[c]	f/f_0[d]
ACP	1.28 (84%)	1.42	10,202 (8,849)	1.19
	2.17 (8%)	2.42	22,650	
MadA	3.78 (95%)	4.21	73,624 (39,196)	1.51
MadA•$(ACP)_2$	1.21 (42%)	1.35	14,624	1.59
	2.34 (10%)	2.61	39,084	
	4.14 (48%)	4.61	91,990	
$MadA^N$	1.78 (88%)	1.99	20,361 (20,622)	1.35
$MadA^C$	2.51 (98%)	2.80	35,211 (18,032)	1.39
MadB	2.40 (99%)	2.68	35,969 (18,428)	1.41

[a] Sedimentation coefficient taken from the ordinate maximum of each peak in the best-fit *c(s)* distribution at 20°C. Percentage of total absorbance present in the selected peak is given in parenthesis. Units are Svedberg (S).
[b] Standard sedimentation coefficient ($s_{20,w}$-value) in water at 20°C. Units are Svedberg (S).
[c] Molar mass values taken from the *c(s)* distribution that was transformed to the *c(M)* distribution. Value in parenthesis is the predicted molecular weight for a monomer based on the amino acid sequence of the protein. Units are Dalton (Da).
[d] Best-fit weight average frictional ratio values $(f/f_0)_w$ derived from the *c(s)* distribution.

Table S2. Distribution of bacterial Mad proteins

Phylum	MadB	MadA	Total
Proteobacteria	406	269	675
Alpha	27		
Beta	41	1	
Delta	11		
Epsilon	11		
Gamma	314	268	
Zeta	1		
Unclassified	1		
Verrucomicrobia	20		20
Firmicutes	11		11
Chloroflexi	7		7
Cyanobacteria	7		7
Bacteroidetes	5		5
Chlamydiae	5		5
Actinobacteria	2		2
Armatimonadetes	1		1
Balneolaeota	1		1
Chlorobi	1		1
Fusobacteria	1		1
Planctomycetes	1	1	2
Spirochaetes	1		1
Unclassified bacteria	1		1
**Total**	**470**	**270**	**740**

Table S3. Relevant protein characteristics of MadB mutant proteins

Protein	Stoichiometry[a]	T_{agg} ± SD[b]	SA ± SE[c]
MadB	dimer	75.47 ± 0.03	132 ± 11
MadB(N43A)	dimer	71.32 ± 0.08	7.9 ± 0.2
MadB(N45A)	dimer	74.84 ± 0.03	0.65 ± 0.02
MadB(H47A)	dimer	73.39 ± 0.03	4.4 ± 0.1
MadB(T49A)	dimer	80.14 ± 0.07	3.8 ± 0.2
MadB(S54A)	dimer	77.86 ± 0.03	40 ± 2
MadB(D79A)	dimer	71.84 ± 0.05	173 ± 6

[a] determined by analytical gel filtration
[b] determined by nanoDSF (T_{agg}, aggregation onset temperature; SD, standard deviation)
[c] determined by malonyl-CoA decarboxylase assay as described in Experimental Procedures (SA, specific activity (pmole/min/pmol protein); SE, standard error)

Table S4. SEC-SAXS-derived properties of MadA and MadB.

Protein	*Rg*[a]	Dmax[b]	MW[c]	Res[d]	RSC[e]	Rg[f]
MadA	38.21 ± 0.11	139.0	85.7	43.0	0.879 ± 0.024	42.44
MadB	23.84 ± 0.04	90.0	33.8	27.0	0.941 ± 0.012	26.43

a
Radius of gyration ± standard deviation in Å derived from the Guinier analysis
[b] Maximum particle dimension in Å derived from the pair distance distribution function.
[c] Expected molecular weight in kDa calculated from a Bayesian inference approach.
[d] Fourier Shell Correlation Resolution in Å of the reconstruction.
[e] Mean real space correlation ± standard deviation of the reconstruction.
[f] Radius of gyration in Å derived from the reconstruction.

Table S5. Bacterial strains used in this study

Strain	Description	Source
NR754	Wild type *E. coli* - *araD*$^{+}$ revertant of MC4100	Dr. N. Ruiz[a]
NR1769	NR754 Δ*fabH*::*kan*	Dr. N. Ruiz
NR754/pBad	Empty pBad in NR754	This study
NR1769/pBad	Empty pBad in NR1769	This study
NR1769/pFabH	NR1769 with FabH expressed from pBad	This study
NR1769/pMadA	NR1769 with MadA expressed from pBad	This study
NR1769/pMadA^{N}	NR1769 with MadA^{N} expressed from pBad	This study
NR1769/pMadA^{C}	NR1769 with MadA^{C} expressed from pBad	This study
NR1769/pMadB	NR1769 with MadB expressed from pBad	This study

[a] Yao, Z., Davis, R. M., Kishony, R., Kahne, D., and Ruiz, N. (2012) Regulation of cell size in response to nutrient availability by fatty acid biosynthesis in *Escherichia coli*. *Proc. Natl. Acad. Sci. U. S. A.* **109,** E2561-E2568. https://doi.org/10.1073/pnas.1209742109

Table S6. Plasmids used in this study

Plasmid	Description	Source
pET28a	IPTG inducible protein expression in *E. coli*	Novagen
pPJ604	MadA in pET-28a	This study
pSGW8	MadA(M17A) in pET-28a	This study
pSGW9	Amino acids 17-329 of MadA in pET-28a	This study
pPJ605	Amino acids 1-161 of MadA in pET-28a	This study
pPJ606	Amino acids 171-329 of MadA in pET-28a	This study
pPJ612	MadB in pET-28a	This study
pPJ614	MadB(N43A) in pET-28a	This study
pPJ617	MadB(N45A) in pET-28a	This study
pPJ615	MadB(H47A) in pET-28a	This study
pPJ616	MadB(S54A) in pET-28a	This study
pPJ619	MadB(T49A) in pET-28a	This study
pPJ618	MadB(D79A) in pET-28a	This study
pPJ602	GlmU in pET-28a	This study
pBad-Myc-HisA	Arabinose inducible expression in *E. coli*	Invitrogen
pFabH	FabH from *E. coli* in pBad	This study
pMadA	MadA in pBad	This study
pMadA^{N}	MadA^{N} in pBad	This study
pMadA^{C}	MadA^{C} in pBad	This study
pMadB	MadB in pBad	This study

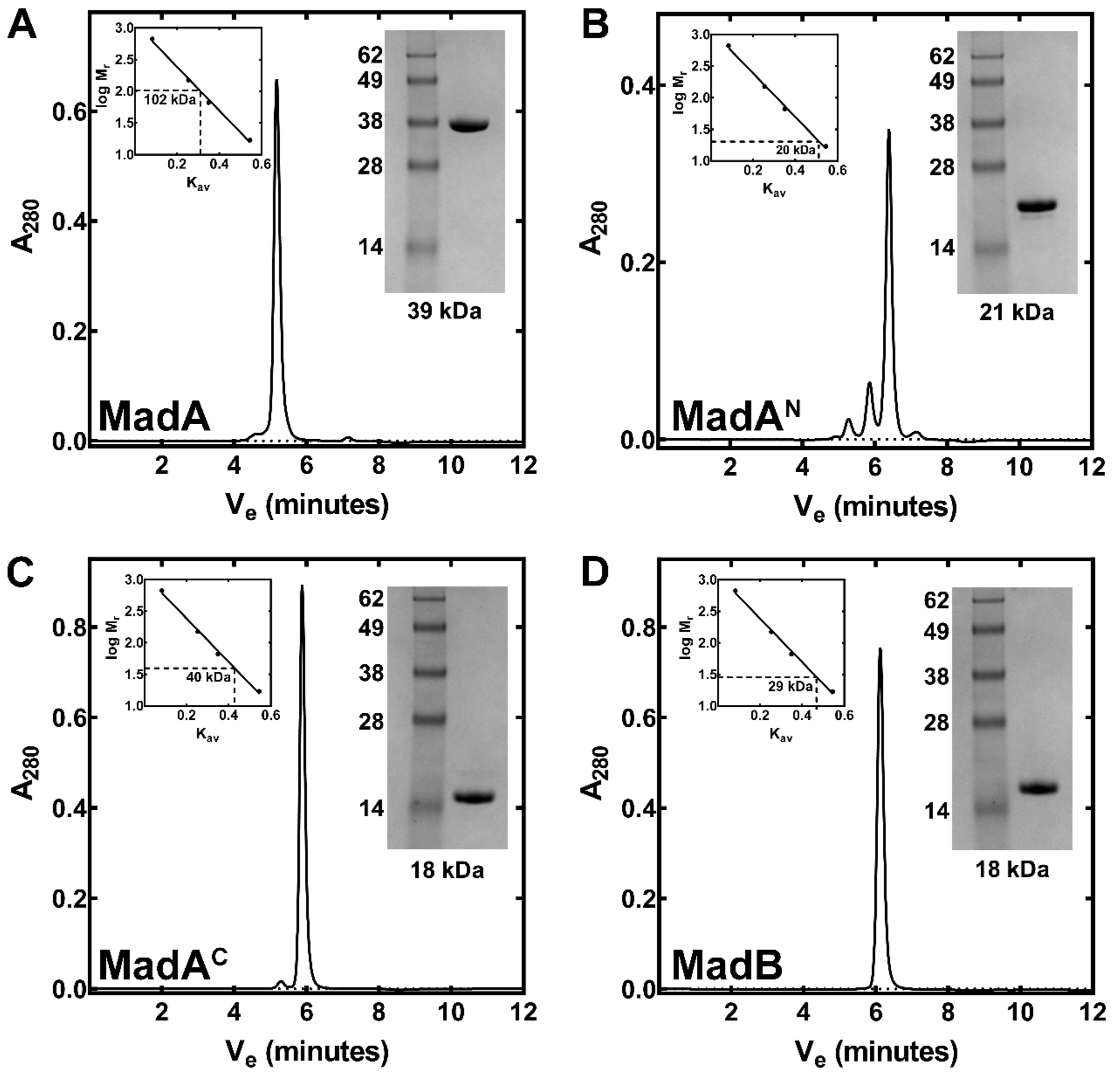

Figure S1. Purity of Mad proteins used in this work. Proteins were expressed as His-tagged proteins and purified by Ni^{2+} affinity and gel filtration chromatography on Sepharose S-200. The calibration curves for the column and an SDS gel showing protein purity are shown as *insets*. *A*, pure MadA elutes as a dimer. *B,* pure $MadA^{N}$ migrates as a monomer. *C*, Pure $MadA^{C}$ migrates as a dimer. *D*, Pure *S. oneidensis* MadB migrates as a dimer.

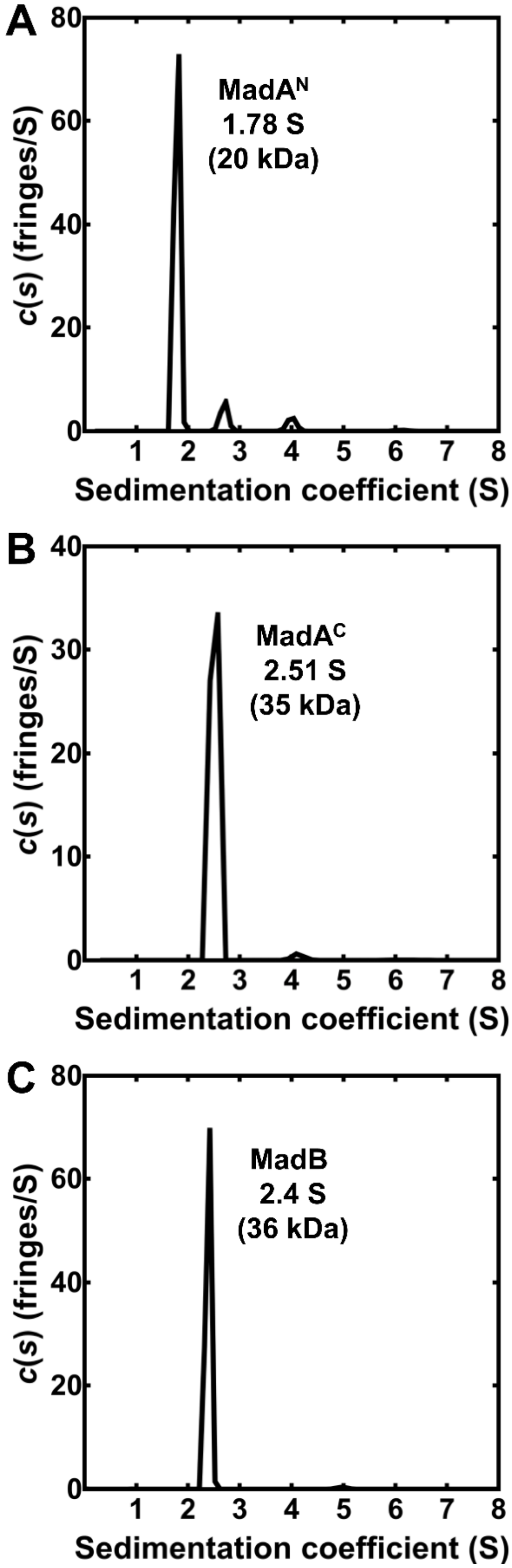

Figure S2. Sedimentation analysis of Mad proteins. A series of sedimentation equilibrium analyses were performed to determine the molecular dimensions and oligomerization state of the various Mad proteins. *A*, sedimentation analysis of MadA^{N} identifies it as a monomer. *B*, sedimentation analysis identifies MadA^{C} as the dimerization domain of MadA. *C*, sedimentation analysis verifies *S. oneidensis* MadB is a dimer. Sedimentation equilibrium parameters are listed in Table S1.

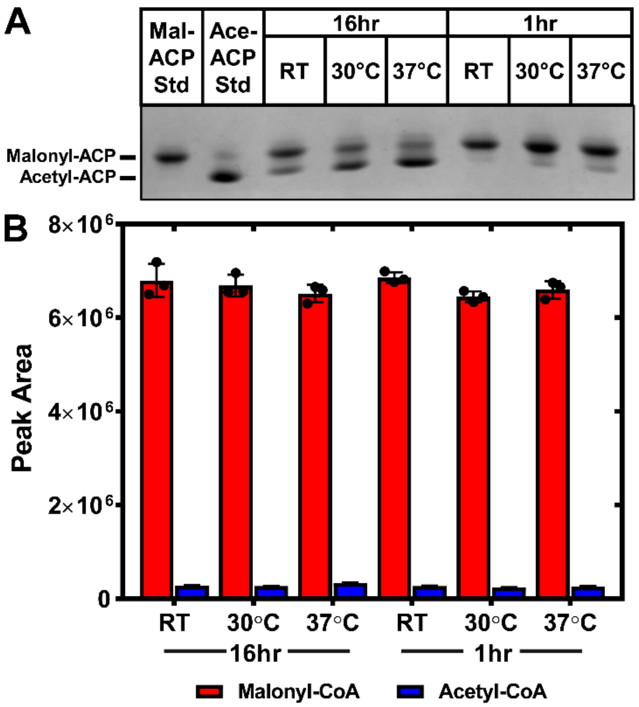

Figure S3. Stability of [^{14}C]malonyl-ACP and malonyl-CoA. In both experiments, the samples were incubated in Bis-Tris, pH 6.0, and samples were incubated for the indicated time and temperature. *A*, instability of [2-^{14}C]malonyl-ACP. [2-^{14}C]Malonyl-ACP was prepared using AcpS, and the [^{14}C]malonyl-ACP and [*14*C]acetyl-ACP were separated by urea gel electrophoresis. *B*, malonyl-CoA and acetyl-CoA were separated and quantified by HPLC by following absorbance at 260 nm.

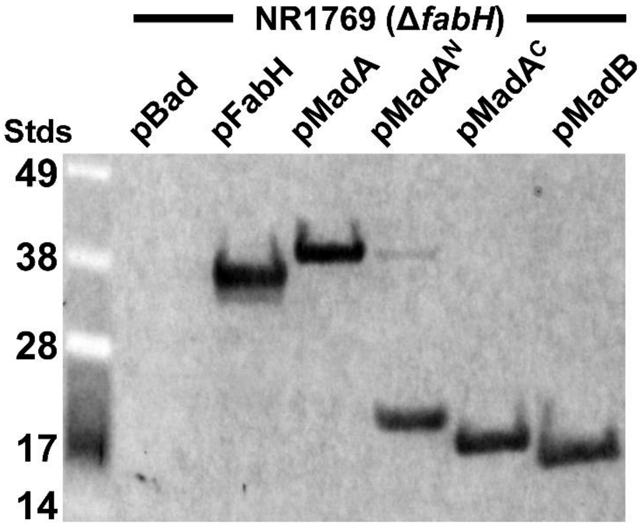

Figure S4. Expression of His-tagged Mad and FabH proteins. Strain NR1769 (Δ*fabH*) was transformed with either the empty pBAD vector or plasmids with the indicated genes under control of the arabinose-inducible promoter. Samples of strain NR1769 (ΔfabH) grown with arabinose were obtained during the complementation experiments outlined in Figure 6 as a control to verify protein expression. The gels were immunoblotted with anti-His-tag antibody as described under Experimental Procedures to detect the expressed proteins.

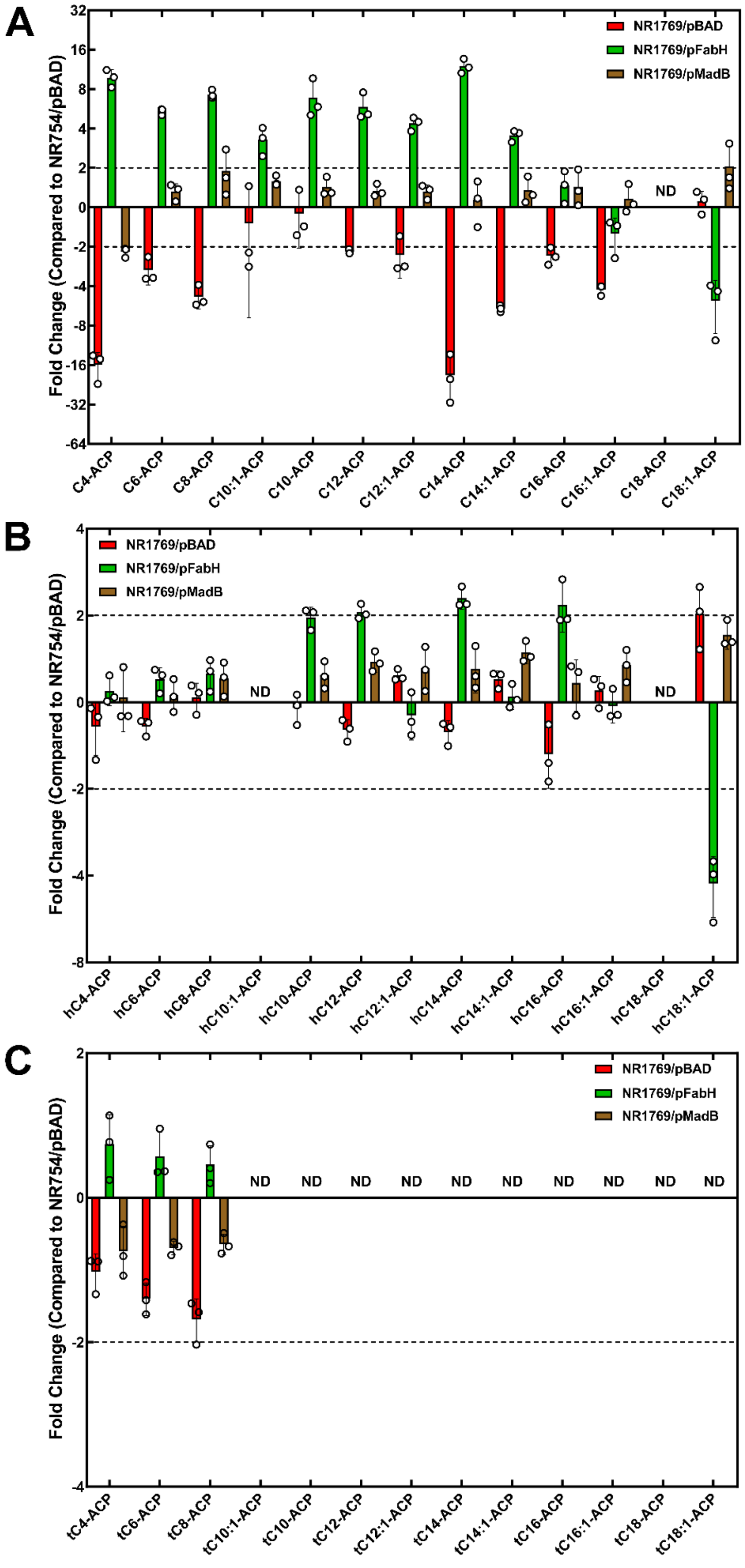

Figure S5. Acyl-ACP pool composition in strain NR1769 (Δ*fabH*) and its complemented derivatives. The acyl-ACP pools were measured by mass spectrometry as described under Experimental Procedures. The individual peaks were normalized to the [$^{13}C_2$]acetyl-ACP internal standard, and the relative abundance of each peak was compared to the wild-type strain NR754. Triplicate biological replicates were analyzed. *A*, relative abundance of acyl-ACPs. *B*, relative abundance of 3-hydroxyacyl-ACPs. *C*, relative abundance of *trans*-2-enoyl-ACPs. There were no ketoacyl-ACPs detected in these experiments. ND means not detected.

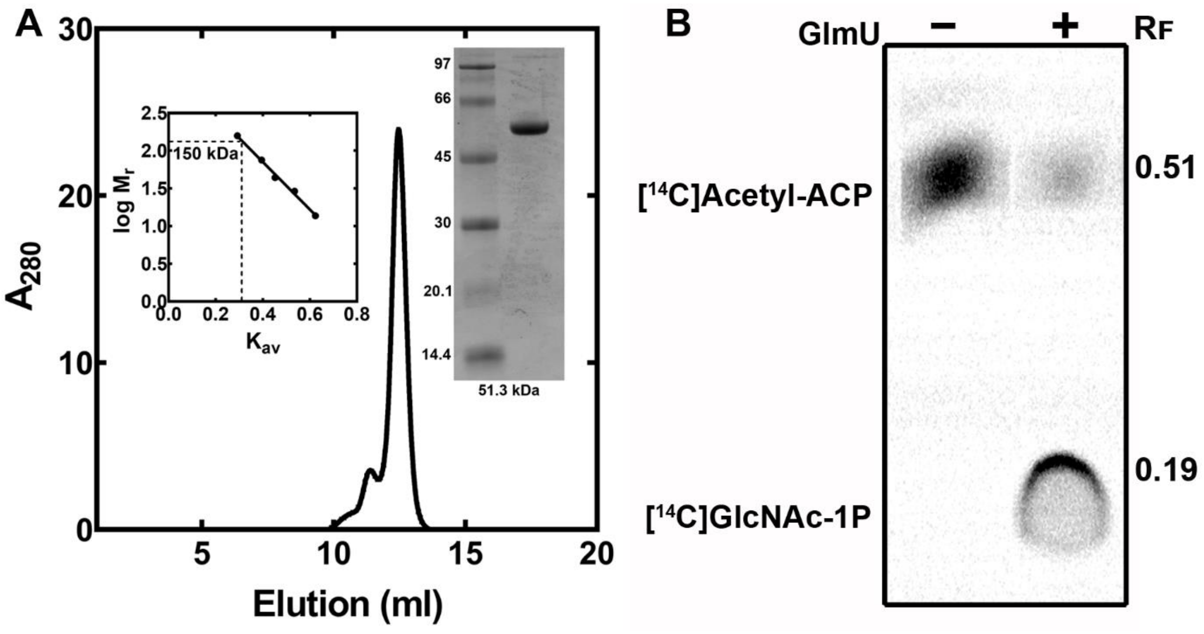

Figure S6. Purification and GlmU assay with [^{14}C]acetyl-ACP. GlmU was expressed as His-tagged protein and purified by Ni^{2+} affinity followed by gel filtration chromatography on Sepharose S-200. *A*, gel filtration chromatography of pure GlmU. The calibration curve for the column and an SDS gel showing protein purity are shown as *insets*. *B*, thin-layer chromatography on Silica Gel H plates developed with 1-butanol:methanol:ammonia:water (5:4:2:1, v/v/v/v) of a GlmU assay using [^{14}C]acetyl-ACP as substrate.
